# Perspectives and presentation of mental health among women from rural Maharashtra (India): A qualitative study

**DOI:** 10.1017/gmh.2024.28

**Published:** 2024-03-06

**Authors:** Pooja Gala, Arunima Ticku, Tanvi Pawar, Shivani Sapre, Pooja Gupta, Kaavya Iyer, Hansika Kapoor, Rupa Kalahasthi, Savita Kulkarni, Poorvi Iyer

**Affiliations:** 1 Ananya Birla Foundation, Mumbai, India; 2 Department of Psychiatry, National institute of Mental Health and Neurosciences, Bangalore, India; 3 Department of Psychology, Monk Prayogshala, Mumbai, India; 4 Rochester Institute of Technology, Rochester, NY, USA; 5 Gokhale Institute of Politics and Economics, Pune, India; 6 London School of Economics and Political Science, London, UK

**Keywords:** mental health, rural India, women, stigma, discrimination

## Abstract

**Objectives:**

A significant gap is observed between the proportion of individuals suffering from mental health (MH)-related conditions and those receiving adequate MH care services, especially in rural areas. This study highlights and contextualizes MH concerns and its extant knowledge as well as gender roles in rural Maharashtra (India).

**Methods:**

Using in-depth interviews, MH themes were highlighted analytically among 72 female beneficiaries of Svatantra from the six administrative divisions (Konkan, Nashik, Pune, Aurangabad, Amravati and Nagpur) in the state of Maharashtra, India.

**Results:**

The notion that MH concerns exist among women from rural communities was well supported. Along with MH concerns, the participants reported somatic concerns in the context of adverse life experiences. Furthermore, systemic issues such as financial problems, familial concerns, presence of addictions and pressures of gender role-related responsibilities were significant triggers for MH problems.

**Conclusions:**

Overall, this study aimed at improving the understanding of the MH needs of women in rural Maharashtra, which can further catalyze an exploration of their general MH and devise suitable interventions for the same.

## Graphical abstract

**Figure:**
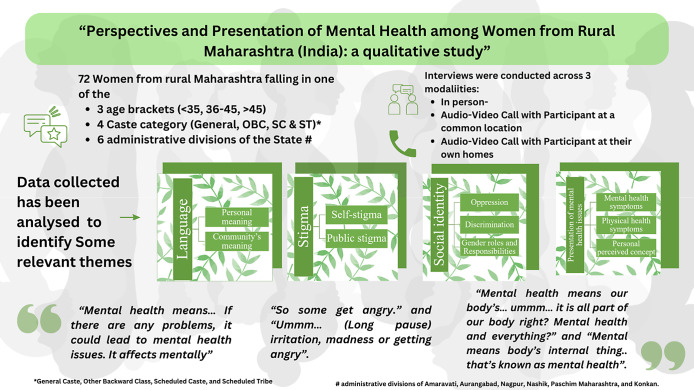

## Impact statement

This research unravels the unique narrative of mental health (MH) concerns among women in rural Maharashtra, India. It highlights how MH concerns are understood by these women. It further emphasizes that the role of systemic stressors such as financial problems, disputes at home and one’s social identity in relation to one’s gender add to the MH burden. Furthermore, self-stigma was highlighted in the form of perceived incompetence in responding to the interview questions. This finding could potentially indicate a gap in the awareness around MH concerns and their treatments. We also found that the lines between physical health and MH are blurry, with the former often being used to understand the latter. We contextualize the extant literature of MH in India and also create a shared language for the same. The findings of this study can be factored into policies regarding MH in rural India, especially as the language around MH and its presentation differ in the rural parts of the country. Thus, MH programs may need to build capacity to effectively address these concerns through mediums that are most acceptable by the rural population if any tangible change is to be seen in rural MH.

## Introduction

India launched its first National Mental Health (MH) Policy in 2014, revised with the Mental Healthcare Act, 2017. A key objective of this act was to provide equitable, affordable and universal access to MH care in India (Sagar et al., 2020). Despite these provisions, there have been notable gaps in equitable access to MH care across India. One such marginalized community is that of women for whom MH services remain a remote reality (Malhotra and Shah, 2015). Gawai and Tendolkar (2019) have highlighted that patriarchal domination in most parts of India, along with low educational levels, poverty and financial dependence of women on men, makes it difficult for women to seek health services. Furthermore, they identify ignorance, misinformation and discrimination as barriers for women seeking MH services.

**Figure:**
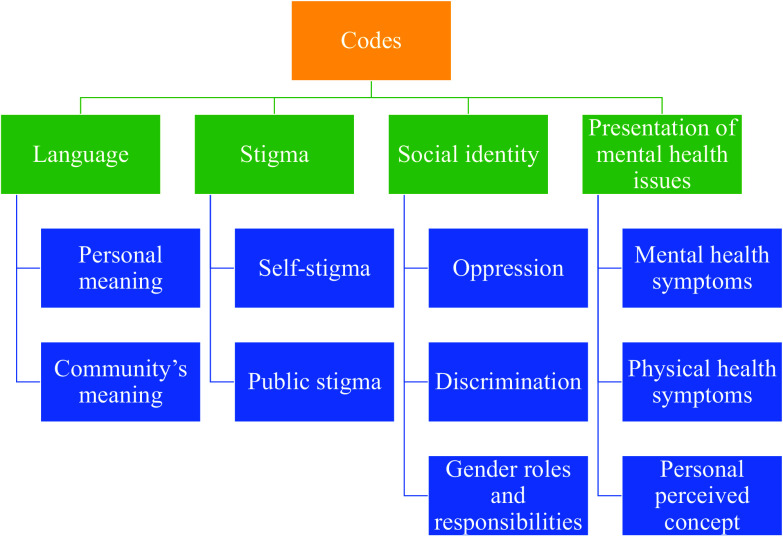

Gender has been known to influence people’s power over their health determinants, including their socioeconomic status and access to essential resources in society. This form of gender disadvantage is observed in areas such as intimate partner violence, lack of autonomy in decision-making, lack of support and bearing children during adolescent age. This is more widespread in rural areas where women are confined to their traditional gender-specific roles such as being a wife, mother and caretaker of their families (Chandra et al., 2020).

Furthermore, one’s gender identity can serve as a disadvantage regarding MH-related conditions in rural women owing to violence, prejudices and lack of privilege (Chandra et al., 2020).

Community- and clinic-based studies in India by Rao et al. (2011) have shown that women are twice as likely to be affected by depression as men. Potential contributors (that are often under-recognized) to this increased gender vulnerability are poverty, social class, marital and childbearing roles, lack of education and continued social oppression. Women tend to not seek help because of stigma, poverty, paucity of awareness or access to care. Even under conditions where they do access care and are prescribed medications, they drop out of treatment due to side effects or the costs of continuing treatment. Thus, psychological distress poses a major economic burden on poor women. Apart from gender identity, in India, explanations for mental disorders are often influenced by systems of traditional medicine and supernatural beliefs. This, in turn, has an impact on the discriminating attitudes that affect both help-seeking behaviors and the quality of care available (Shidhaye and Kermode, 2013).

In addition to one’s gender identity and prevalent explanatory models, social disadvantage, which can come from one’s caste or religious identity, also impacts MH outcomes and help-seeking behaviors. Social disadvantage has been found to be associated with economic disparities, employment outcomes, physical health and access to health care (Gupta and Coffey, 2020). Johri and Anand (2022) have also highlighted that general caste individuals report higher life satisfaction and well-being as compared to the Scheduled Caste (SC) individuals in India. Furthermore, there have been notable gaps reported on self-reported MH indicators between higher caste Hindus and two marginalized social groups, that is, SC and Muslims (Gupta and Coffey, 2020). Given this, the study aimed to provide space to the voices of women from various socially marginalized groups to share their lived experiences.

According to the National MH Survey, about 150 million Indians need care for mental disorders and about 10% suffer from common mental disorders (CMDs) such as depression, anxiety, emotional stress, suicide risk and substance use (Kallakuri et al., 2018). Specifically, in Maharashtra, Shidhaye et al. (2016) in their study have reported how MH concerns in the rural communities are affected by gender and age. Further studies report a prevalence of MH concerns between 8% (Sambutwad et al., 2020)-14.6% (Shidhaye et al., 2016) of the population in rural Maharashtra.

Therefore, the study aims to examine the narrative of MH among women in rural areas of Maharashtra, and their lived experience in terms of prevalent attitudes, discourses, notions and stigmas, regarding MH along with their existing language base to understand the spectrum of MH and illness. The study also aims to explore gender roles and it’s impact, particularly how gender roles interact with MH concerns on the women in rural Maharashtra.

## Methods

The target sample for this study were women entrepreneurs associated with Svatantra who have completed 2 to 3 loan cycles, each cycle lasting a year. This long association provided researchers with the dependable contact details of the participants residing in distant rural parts and a reasonably good response rate due to trust among officials of Svatantra who accompanied and introduced the interviewers to the participants. This led to the smooth facilitation of this study amidst the COVID-19 pandemic from December 2021 to March 2022. Admittedly, women entrepreneurs differ qualitatively from the general section of the female population in rural parts in terms of their agency, financial independence, mobility and access to social capital, among others.

Svatantra has branches in 30 districts of Maharashtra catering to more than 2,30,000 female entrepreneurs. The primary stratification criterion to choose sample districts was the administrative division of the state.^1^ Participants from each selected branch were categorized into three groups (<35 years, >45 years and those between 35 and 45 years old; Table 1). The identified participants were approached for their consent by the field officers (FOs).

**Table 1.** Caste and age-wise distribution of obtained versus targeted sample

	Caste				
Age	General	OBC	Other	SC	Grand total
<35 years	8 (6)	9 (6)	6 (6)	6 (6)	29 (24)
>45 years	5 (6)	7 (6)	5 (6)	6 (6)	23 (24)
36–45 years	4 (6)	6 (6)	5 (6)	5 (6)	20 (24)
Grand total	17 (18)	22 (18)	16 (18)	17 (18)	72 (72)

*Note: Targeted sample in brackets.*

Majority of the clients belonged to the Other Backward Classes (OBC) and general caste categories, followed by the SC category. The proportions of clients from Scheduled Tribes (ST) and other caste groups were clubbed together while retaining the earlier three groups. Three women entrepreneurs were chosen from each of the four caste groups, providing us with twelve women from each branch. Thus, the final sample consisted of 72 women respondents from the most populous branches of all 6 administrative regions (Table 2).

**Table 2.** Sample demographics and interview modalities

Region	Participants	Audio-video	In-person	Audio-video
Amravati	12	1	0	11
Dhule	12	4	0	8
Konkan	13	0	13	0
Nagpur	12	0	0	12
Nanded	12	3	0	9
Paschim Maharashtra	11	0	11	0
Grand total	72	8	24	40

The three interview modalities were explored: a) in person interviews where both the interviewer and the participants were present in person; b) audio-video mode where the participants were present at the Svatantra branch office and c) audio-video mode where participants participated from home or nearby area (Table 2).^2^ The interviews were conducted in Marathi after taking participants’ appointments. The FOs were present to facilitate the interviews while ensuring that they respected the privacy of the interviewed participants.

### Sample selection

As a part of pilot study, a focus-group discussion (FGD) (Supplementary material A) was conducted with six women clients of Svatantra from the Thane district of Maharashtra. The participants were recruited by Svatantra FOs. The participants recruited from general, OBC, SC and ST sections were classified into three age-based categories: <35, >45 and 36–45 years.

The FGD, conducted at the Svatantra Thane office, was an hour long, adhering to the COVID-19 protocols. An early career MH professional (with a Master’s in Clinical Psychology) facilitated the FGD, with two other early career MH professionals present on a video call to record behavioral observations and group dynamics. The participants were asked questions aiming to address the aforementioned five research questions. The participants were then debriefed about the project and were compensated for a day’s wage (in kind through a mobile phone voucher worth INR 300). They were also compensated (in cash) for their travel to and from the Svatantra office before they left the office. The FGD was audio-recorded with verbal consent of the participants.

The procedure and compensation scheme for personal interview (Supplementary material B) was the same as in the FGD. The participants were then debriefed and thanked for their participation.

During the interview, the interviewers rated the participants’ behavior on a scale of 1 (least) to 5 (most). This behavior checklist (Supplementary material C) was used to assess the participants’ nonverbal behavior. For both the FGD and individual interviews, a distress protocol (Supplementary material D) and emotion regulation skills (Supplementary material E) were curated for use in case of any triggers in any participant. Participants were equipped with a list of telephonic MH resources (Supplementary material F). Out of the 72 interviews that were conducted, data saturation was reached by 36 interviews at which point further coding was stopped.

### Coding scheme

As in deductive thematic coding steps, a codebook (Supplementary material G) was developed using the top-down coding, also known as the “a priori” approach. Specifically, the overarching themes of interest were mapped (based on extant literature and results from the FGD and initial interviews) before starting the analysis (Attride-Stirling, 2001).

Based on the thematic networks’ technique (Attride-Stirling, 2001), this codebook has a larger global theme, which posed as research questions in the codebook. These were further bifurcated into organizing themes that posed as answers to these questions (characterized by inclusion criteria and definition). In the process of coding the first nine interviews, the inclusion criteria were reviewed and revised to bring in unanimity among coders on the said organizing theme. The codebook (see Supplementary material E) was divided into four global themes:

#### Language (organizing themes: Personal meaning and community meaning)

The global theme of language was created to understand the existing language base for MH-related concerns among women in rural Maharashtra. This was divided into two organizing themes: personal meaning and community meaning. Personal meaning refers to an individual’s perception of MH, and community meaning refers to the language used by the community at large to understand MH-related matters. Narayan (2013) has reiterated the need to create better mechanisms that allow medical students and health workers to obtain a functional knowledge of languages they encounter in the clinic, to promote the efficacy of interventions provided. The cultural narrative that one is a part of is heavily influenced by the shared language that one uses to understand oneself and others around (Narayan, 2013).

#### Stigma (organizing themes: Self-stigma and public stigma)

The global theme of stigma was created to understand how negative societal reactions toward people suffering from MH-related concerns shape their perception of MH. This was divided into two organizing themes: self-stigma and public stigma. Self-stigma refers to individual members’ negative attitudes and perceptions toward MH (Arvind et al., 2019). Public stigma refers to an individual’s beliefs regarding the attitude of others in the community toward people with MH-related concerns. Existing research has shown that stigma prevents affected people and their families from admitting to or accepting the MH-related condition and thus reduces help-seeking behavior, which in turn results in a treatment gap (Arvind et al., 2019).

#### *Social identity* (*organizing themes: Discrimination, oppression and gender roles and responsibilities)*

The global theme of social identity was created to understand how adherence to social labels such as caste, religion, gender, and socioeconomic status impacts an individual’s perception of their MH-related concerns. This was divided into three organizing themes: oppression, discrimination and gender roles and responsibilities. Oppression refers to the subjugation of a person or group of people either emotionally or physically due to the receiving party’s social caste/status/gender/political or religious affiliation.

Discrimination refers to the unfair treatment of an individual or a group on account of belonging to social groups such as those based on caste, gender, age and religion. Gender roles and responsibilities indicate an individual’s impact of perceived gender roles and responsibilities on their own MH and that of others.

There is a wealth of psychological research outlining how discrimination can exacerbate stress and that discrimination-related stress is linked to MH issues, such as anxiety and depression, especially in vulnerable groups such as women and children (Sirin et al., 2015). Perceived discrimination has also been linked to specific types of physical health problems, such as hypertension, self-reported poor health and breast cancer, among women (Pascoe and Smart Richman, 2009). Research by McGibbon and McPherson (2013) highlighted that violence against women is one of the key factors for women’s stress across the life course and that oppression has a profound and long-lasting impact on the body’s stress handling system. Hence, it would be crucial to explore the intersection of one’s gendered social identity coupled with other variables such as caste, religion, and so forth on one’s perception of MH-related concerns.

#### Presentation of MH issues (organizing themes: Antecedents, MH symptoms, physical health symptoms and perceived personal concept)

Presentation of MH issues as a global theme was created to understand the various triggers, mental and physical health-related symptoms, and one’s personal perceived concept in the context of MH and related concerns.

Malhotra and Shah (2015) highlighted that patterns of psychiatric disorders and psychological distress among women are different from those seen among men. Furthermore, symptoms of depression, anxiety and unspecified psychological distress are two to three times more common among women than men. It has also been suggested that observed gender differences in the prevalence rates originate from women showing a higher tendency to internalize their difficulties.

Thus, the perceived concept of themselves (personal perceived concept) based on their various life experiences (antecedents) have an impact on their mind and body (mental and physical health-related symptoms) was deemed appropriate to explore.

### Coding procedure

#### Pilot

The recordings of the interview were transcribed into English by a translation + transcription service provider. The transcribed interviews were then coded by two coders who are fluent in English. Both coders hold a Master’s degree in Psychology. In this pilot round, three interviews were coded first, with the purpose of identifying gaps in the codebook (if any), and also to streamline the coding process.

The first interview was coded on Taguette – an online software for coding qualitative interviews. Thereafter, Excel was used for the rest of the coding. On Excel, entire responses were coded, and multiple codes were often given to the responses that encompassed various themes. The codebook template was loaded onto Excel, and if a particular response fit that code, it was marked as 1 (0 is not), thus contextualizing the codebook.

#### Final

The next phase involved coding six more interviews in pairs to check for consistency among coders. That is, two pairs coded three interviews each. These were a fresh set of coders of which one was also involved in creating the Codebook, and the other three were interviewers. The former was then replaced with another coder who was trained in coding and the Codebook. Kappa’s coefficient was used to establish the inter-rater reliability (IRR). This was done to ensure that the coders align on their understanding of each code. This was an iterative process wherein, upon finding low IRR scores, the coders would discuss the discrepant responses (responses coded differently by each coder), and arrive at a shared understanding of the codes (for an extensive overview of the process, see Supplementary material H). Table 3 presents the IRR values across the nine interviews, yielding an average IRR of 0.73 across all global themes (also see Table 4).

**Table 3.** Interview guide

*Section 1: Shared language (to understand individual/group perception of mental health and illness)*
What is mental health according to you? What does community mean to you? What does mental health mean in your community? Is mental health discussed in your community? If yes, how? Can you describe a mentally healthy person in your community? What does being mentally unwell mean in your community? What are some of the words used to describe people who might be mentally unwell? Can you tell me about a recent time when you were upset? Did you experience any body symptoms such as ache in your head, stomach, back or chest when you were upset? Do you think the two are connected? Have you observed if women in your community report similar aches or pains when they feel upset? How do women in your community express negative emotions such as feeling unhappy/sad/angry/upset/scared and so forth? Have you observed that people who are mentally unwell are treated differently? If yes, how?

*Section 2: Understanding gender roles and existing structures (exploring the existing systemic narrative into account)*
What common experiences impact the mental health of women in your community? What kind of work related responsibilities affect the mental health of women in your community? How? Does having specific roles and responsibilities such as managing work/business, house expenses, taking care of children, taking care of elderly, cooking and so forth affect the mental health of women in your *house*? How?

**Table 4.** Inter-rater reliability for each code

		Pearson’s *R*	Kappa	ASE	Mean IRR
Invalidity	(In)validity of response	0.903***	0.903***	0.023	0.90
Language	Personal meaning	0.671***	0.666***	0.059	0.67
	Community meaning	0.671***	0.671***	0.05	
Stigma	Self–stigma	0.844***	0.832***	0.117	0.78
	Public stigma	0.725***	0.72***	0.071	
Social identity	Oppression	0.816***	0.799***	0.143	0.73
	Discrimination	0.584***	0.583***	0.143	
	Gender roles	0.821***	0.816***	0.06	
Presentation of MH issues	Antecedents	0.77***	0.77***	19.57	0.71
	MH symptoms	0.776***	0.77***	0.046	
	Physical health symptoms	0.676***	0.675***	0.064	
	Personal perceived concept	0.643***	0.643	0.062	

## Results

### Demographics

Out of 72 interviews, 50% of women were educated below the 10th grade, followed by 16.6% till the 12th grade, 19.4% till the 10th grade, 4.16% up to graduation and 2% up to postgraduation, and 1.38% reported ‘other’ and no schooling. Then, 38 women belonged to joint families and 34 to nuclear families, with an average of 5.5 people in the household. There was an average of 1.28 children under 18 years and 1.23 children above 18 years. With regard to religion, most women practiced Hinduism (82%), followed by Buddhism (15.2%), Islam (1.38%) and other (1.38%). Participants were recruited after matching their caste with the caste stratification in Maharashtra. For age- and caste-wise distribution, refer to Table 1.

### Behavior checklist

The participants were rated on nonverbal aspects during the interview, including their response patterns to MH-related questions. Data indicate that most women did not hesitate much when talking about MH as most were scored on the lower end for the refusal to talk (*M* = 1.17), the need of probes (*M* = 3.01), tangentiality (*M* = 1.65) and changing the topic (*M* = 1.17). Therefore, it can be inferred that women from rural Maharashtra are generally open to conversations on MH concerns.

## Qualitative research findings

The global theme of language was created to answer the research question of “How do people talk about MH in rural Maharashtra?” Personal meaning, which consisted of 13 basic themes, was coded more frequently than community meaning. This could imply that participants are more attuned to understanding their own meaning of MH rather than how society describes or views MH. Extracts for each basic theme are highlighted in Supplementary material I.

Out of these, the basic themes that received the higher responses are the presence of problem, stress or tension; anger and aggression and physical and MH.

The presence of problems, stress or tension can be best explained in terms of mental illness being understood as a result of one having ‘‘problems’, ‘stress’ among women in rural Maharashtra. A chief finding under this basic theme was terms such as problems, stress or tension were words that were consistently used to describe poor MH.

An outburst of anger, a bad temper or aggressive tendencies were also terms used as signs of mental illness.

We also find that physical and MH are intertwined, that is, MH is understood in the same context as being the same as physical health. This shows how lines are blurred when it comes to categorizing symptoms related to physical and MH.

Under the global theme of presentation of MH issues, MH symptoms were the one with the highest number of responses coded to it. This organizing theme contained reflections that were in concordance with the research question, that is, “In rural Maharashtra, what are the current MH needs of women?”

From the responses analyzed, 17 basic themes were extrapolated. In our analysis, somatic concerns presented themselves in participant accounts. Participants discussed bodily concerns such as headaches, backaches, migraines, chest ache, restlessness in the body and menstrual symptoms. These somatic concerns manifested in the face of a spectrum of adverse life experiences such as familial conflicts, economic issues and the passing away of a closed one.

Evidence of mental distress was found based on day-to-day stress-provoking situations, which can extrapolate into several behavioral manifestations. It includes stressful situations in life – caused by fights at home, children (refers to discord/conflicts) and everyday mundane familial problems. This theme also managed to throw light on the societal pressures imposed on a woman due to which she became distressed. Out of the four organizing themes, antecedents consist of 16 basic themes that address stressors ranging from financial troubles and addiction to spousal disagreements and work conditions.

Financial troubles have been operationally defined as a lack of money, financial instability and stress from loans. The second basic theme is titled presence of addiction within family members. Women reported that having a family member who suffers from alcohol addiction as a major stressor and a direct link to their own MH concerns. Participants described ‘problems within their families’ (the third basic theme) such as lack of support from family members, harassment and mistreatment by family members and arguments and disputes within the family as stressors and antecedents. Spousal disagreements, lack of emotional support from immediate family members, domestic abuse or harassment and lack of financial support and encouragement from family members are causes of daily stressors.

The global theme of social identity had an organizing theme of gender roles with the most responses. The two research questions attributed to this global theme were, “in rural Maharashtra, what are the gender roles maintained by women?” and “in rural Maharashtra, how do these gender roles impact the MH of women?”

Out of the nine basic themes that were drawn out, three had a significant number of responses attributed to them. The first of these three themes were the ‘burden due to household chores’. This theme was described as the feeling of strain on physical and MH resources due to a large number of household responsibilities without substantial help from a spouse or other family members. This theme was constructed out of the responses pointing toward a burdensome load of responsibilities imposed on a woman because of a systemic belief that is very natural for a woman to take a caregiving role.

The next basic theme was the burden felt due to work responsibilities, which was indicative of a feeling of strain on physical and MH resources due to burdensome work responsibilities that are supposed to be fulfilled due to economic conditions. The responses that were coded into the theme spoke largely about having to pursue some occupation due to below-par economic conditions, while also fulfilling familial responsibilities. When asked to elaborate on the above aspect and how it had an impact on physical and MH.

This above-mentioned basic theme also indicated their ‘lack of personal choice’ when it came to choosing to pursue an occupation.

Another basic theme closely connected with the above two that also had a significant number of responses coded to it was ‘marital obligations.’ The theme was reported as fulfilling duties imposed by society and family, toward the spouse even while receiving no reciprocity from him. This basic theme can be further interpreted as a socially imposed sense of duty that a woman has to fulfill even though her spouse is not contributing in the household.

Under the global theme of stigma, it was observed that the majority of the participants mentioned that they do not feel they are competent enough to answer the questions about MH as they do not know anything.

Above-mentioned responses point toward lack of exposure and confidence in one’s abilities of the participants. They mentioned not being able to give answers because they lack knowledge and they do not have prior experience of interviewing and stating their opinions.

Some women also mentioned overlooking and not acknowledging the distress they face due to household responsibilities and looking after family members. Some women also mentioned hiding their physical pain and not sharing their stressors with anyone. The lack of confidence contributes to the understanding of self-stigma. Public stigma was assessed to check if there is any interpersonal stigma associated with deviant behaviors of people.

Participants also reported the usage of ‘discriminatory words or language’ toward people who suffer from mental versus physical health concerns. Some of the words stated by participants were ‘mental’ and *‘*mad.’ On the contrary, some participants also outrightly denied people being treated differently because of their conditions.

## Discussion

The purpose of the study was to understand how MH is conceptualized among women in rural Maharashtra. The core research questions covered topics such as understanding of MH (what language is used regarding MH), stigma (internalized and externalized), how MH concerns are manifested (presentation of MH symptoms) and understanding the role of gender to contextualize MH problems (social identity).

As the report of the District Mental Health Program has highlighted, in the 9th plan period, some districts have utilized only 37–47% of resources. They also identified the reason for underutilization to be administrative delay. The report also found that awareness regarding MH symptoms, such as psychosis, neurosis and epilepsy were found very frequently in some districts. Some of the interesting findings from the report also point toward some occult practices that are preferred by some villagers due to an assumption that MH symptoms are caused by evil spirits and they cannot be treated at home (Ministry of health & family welfare, 2016). Women in the age group of 40–59 years have the highest prevalence of CMDs in India; therefore, their thoughts, feelings and experiences around MH are important to understand. Women in India face many health-related issues, including MH issues, which has an overwhelming effect on the population. Despite the higher prevalence of CMDs, they are rarely recognized or provided treatment in time. Prevalence of these CMD symptoms in India counts for one-fifth of the global population; yet, the exact prevalence rate is approximated and real numbers are unknown (Jayasankar et al., 2016).

Expanding on this literature, our qualitative study found that women in rural areas may not necessarily know the technical terminology when it comes to psychological or psychosomatic concerns; however, there is an understanding that MH concerns do exist. As emphasized by Jena et al. (2020), the perspective of people needs to be taken into account along with their location, gender and education when planning the psychiatric healthcare, as their perception/attitude serves as facilitators/barriers for achieving MH care goals. Hence, this study helps in highlighting the prevalent attitudes related to MH care among women in rural Maharashtra.

Prior literature in the discussion of gender and its biopsychosocial correlates has cited evidence of gender and its association with physical pain (Keefe et al., 2000; Latthe et al., 2006). A qualitative study conducted in a rural district of Bihar indicates that the strenuous nature of the work carried out by women, both inside and outside the house was found to be a primary cause of pain. Another analysis was conducted in the forests of Garhwal, discovering ancient practices that make gender, work and pain systemic. The article explored examples of women carrying out tasks that require them to stretch the boundaries of their physical capabilities, and lead to physical exhaustion. These chores are woven into the social identities of these women, which could lead to hardships (Gururani, 2002). These studies collectively implicate a gender-based discrepancy with the nature of work that the women in rural areas carry out. Physically strenuous tasks that are woven into the social identities of these women lead to significant distress such as back pain, although, this pain is often not paid much attention to.

We also understand that the lines between physical health and MH are rather blurry, with the former often being used to understand the latter. This study brought to light that systemic issues such as financial problems, problems within the existing family dynamics, or the presence of addictions act as significant triggers and stressors for MH problems among the participants. Other systemic variables attached to expectations of one’s gender identity, such as the burden of multiple roles women are required to play at work and at home, with little to no personal choice in making decisions with regard to their chores, and the presence of marital obligations, act as added stressors toward perceived MH-related problems.

### Limitations

As India is a culturally diverse nation, other rural areas of India may have nuances not reflective in our current sample. As our participants were Svatantra beneficiaries, this makes the sample more specific due to their unique demographic of having some financial autonomy as against women who primarily identify as homemakers in rural India. Hence, the results of this study should be generalized to a larger women population with caution. Finally, the data collection took place during the COVID-19 pandemic; therefore, a large number of participants were only able to participate via video calls or telephone calls as opposed to in-person interviews, which could have led to the loss of potentially rich nonverbal data. At the same time, factoring in the accessibility of telehealth and using digital mediums for doing qualitative research is one of the boons of conducting such a study during the COVID times.

### Implications for future research

The insights from this study give insight into the kind of MH resources needed in rural India. The shared language developed as a part of this study can be used to provide contextualized corrective psychoeducation. Conducting wide-scale, tailored surveys could help delineate chief MH concerns among women in rural Maharashtra and could facilitate the development of accessible and adaptable interventions. This study can be used to open doors to future research that focuses on empowering, supporting and sustaining quality MH care initiatives and efforts for the betterment of women in rural areas.

## Supporting information

Gala et al. supplementary material Gala et al. supplementary material

## Data Availability

The data that support the findings of this study are available on request from the corresponding author. The data are not publicly available due to privacy or ethical restrictions.

## Footnotes

1 Branches with the highest number of clients were likely to have enough variability in terms of age and caste status. Therefore, branches with the highest number of clients and the corresponding districts were selected from each administrative division, namely, Amravati, Aurangabad, Nagpur, Nashik, Paschim Maharashtra and Konkan.

2 Notably, the pandemic situation constrained the goal of administering a unified number of interviews through each of the modalities owing to untimely restrictions on mobility and transport strikes in rural parts of Maharashtra.

## Supplementary material

The supplementary material for this article can be found at http://doi.org/10.1017/gmh.2024.28.
