# Supplementary material for: Perspectives and presentation of mental health among women from rural Maharashtra (India): A qualitative study
Source: Glob Ment Health (Camb). 2024 Mar 6;11:e40. doi: 10.1017/gmh.2024.28 (PMC11016358; doi:10.1017/gmh.2024.28)
Supplement: Gala et al. supplementary material [file S2054425124000281sup001.docx]

**Supplemental Material A**

**Guidelines for focus group discussions**

**Process:** Consent (individually) - Demographics (individually) - Focus group discussion (group) - Debrief/Closure (group)

### ***General instructions (how to read this document):***

This is a guiding document to help you facilitate the FGD process, and must be used only for internal processes, to understand how the focus group discussions are to be conducted. Specifically, this document highlights the manner in which instructions and questions are to be administered to the participants such as the processes to follow if the participant needs any clarification or direction to respond to the questions. The purpose of this document is to aid a seamless focus group discussion, and avoid any interviewer bias that may influence participant responses.

You will find these terms in this document:

**“GP” = Guiding Probe -** You may use these terms while conducting the focus group discussions. Ensure that these terms are used only to ***guide*** their responses, such that they are not deviated from the question objective. In doing so, the participants must not be primed in any manner.

**“QO” = Question Objective -** This is to help you understand what purpose the proposed question aims at fulfilling. This must ***not*** be used with the participant. At your own discretion, you may use other probes (without priming the participants) only to fulfill the QO.

The probes/meaning statements must only be used in case the participant is unable to understand the questions, is unfamiliar with the terminology used, or asks for any direct clarification. The guiding probes are in no way binding or definitive with regards to how the participant is supposed to respond. The document also lists “Question Objective” for each question with the motive to guide the FGD.

***Instructions for taking Consent:***

The consent form (attached here, and will also be provided to you separately for the FGD) will be read out to the participants to seek their willingness for participation in the study. Please note that the consent is to be taken for each participant individually, and not in a group. The consent form includes the process of the focus group discussion, logistical details on how the focus group discussion will be carried out, the participants’ rights in the process, potential benefits, risks involved, and final verbal agreement to be taken. Please keep the consent-taking process conversational in nature, and make sure that you begin recording the interaction with the participants’ from this point onwards. In doing so, you will have a separate recording for each participant, with their consent and demographics (below-mentioned), and a single (collective) recording with all the participants in the focus group discussion. It is important to reiterate that their participation in every step of the focus group discussion, that their participation is entirely voluntary, and that their relationship with ******* will not be impacted if they choose to stop participating in the focus group discussion at any point.

Following is the consent form (it will also be provided to you separately):

**INFORMED CONSENT FORM**

**Introduction**:

Hi. How are you doing? My name is _____________. I am a (mental health professional) and a research assistant at Ananya Birla Foundation. Firstly, I want to thank you for taking your time out and meeting with us.

**Purpose:**

The Ananya Birla Foundation, Mumbai is doing a project on understanding mental health of women in rural Maharashtra.

Your participation in this study is voluntary and you can withdraw from the study at any point of time without giving any reason. If you have any doubts or feel uncomfortable at any point in the FGD, please let me know.

Please be assured that personal information such as your name and phone number will be kept completely confidential and will be utilized only for research purposes such as coordinating with you. The information collected through such FGDs will be used to write reports that would be published in a scientific journal or presented in a conference after removing your personal details. No identifying information will be mentioned.

*Do you have any questions?*

In this FGD, I will ask you all a few questions and your answers will be audio-recorded. The recordings will be kept safe and not shared with anyone other than the researcher.

*Do you have any questions?*

**Benefits, Risks, and Costs**

You will be given a token of gratitude for participating in this study in the form of a 300 rupees mobile recharge voucher. *(For those who are travelling: your travel to and fro the office will be covered by us)*

During the FGD, you may experience some discomfort or distress when talking about some of your experiences or those of women in your community. If this happens, please let me know and I will address your concerns to the best of my ability. If need be, we will stop the FGD process. I will also guide you to an appropriate support system. After participating in this study, you may want to talk about your mental health more. We will also share some helpline numbers with you, for any emergencies regarding mental health.

I want to assure you that your decision to participate in this study will in no way affect your professional relationship with *_________*.

*Any doubts/clarifications so far?*

**COVID 19 Protocols**: We are taking the necessary precautions for COVID-19 by keeping provisions for masks, hand sanitizers, and maintaining physical distancing when doing the FGD in person and otherwise.

**Verbal Consent (audio recorded)**

Participant:- *“I have been informed that I am being asked to participate in a research which will explore the experiences of mental health of women in my community.*

*I understand that I have been asked to be a participant in the project. I am willing to give my time for the recorded FGD. I have understood how my data will be collected and shared, and that I have the right to refuse my consent or to withdraw from the study at any point, should I like. There are no costs of withdrawing from the study”*

*Researcher: Yes, you are right.*

***Disclaimers: Any point in the focus group discussion, if:***

1. **A participant breaks down** - pause and ask if they are okay to continue, and would like a few minutes to feel better. In case they do not seem okay, please pause the focus group discussion, remove the participant from that setting, and provide them with the list of mental health care resources (appended in the Debrief section). Once the participant feels better, you may continue the focus group discussion with the others.
2. **A participant appears disoriented** - pause and check if they are feeling okay, and would like to continue with the FGD process. In case they do not seem okay, please pause the focus group discussion, remove the participant from that setting, and provide them with the list of mental health care resources (appended in the Debrief section). Once the participant feels better, you may continue the focus group discussion with the others.
3. **A participant does not seem to interact much** - involve them in the discussion by asking them what they think about the topic of discussion, and what their experience has been like.
4. **A participant seems to dominate the discussion** - involve others by encouraging participation. You could say “I want as many people to contribute to this discussion as possible”
5. **The discussion does not seem oriented to the questions** being asked (going off tangent or providing information irrelevant to the question) - Repeat the question as is, and use probes to guide them to respond in line with the QO. Also ask “What do you think is being asked in this question?”

***Instructions for Demographics***

Demographic details need to be taken independently for each client. This needs to be done at the start of the study. For the focus group discussion, please ensure that the other participants are not in the room while recording the consent and demographic details.

**Demographic form (will also be provided to you separately):**

1. Name:
2. Age (in years):
3. Year of Birth:
4. Marital Status: Single | Married | Separated | Divorced | Widowed
5. Educational Level: Below 10th | 10th | 12th | Graduation | Post-graduation | Diploma | Vocational courses
6. Type of family (nuclear/joint):

*(Nuclear -- Living with parents and siblings if unmarried; partner if married)*

*(Joint -- Living with parents, grandparents and siblings if unmarried; living with in - laws, siblings and family if married)*

1. Number of people in your household:-
2. Number of:-

- Children (below 18 years of age):
- Children (above 18 years of age):
- Adults (above 60 years of age):
- Siblings:
- Siblings in law:
- Parents:
- Parents-in-law:
- Grandparents:
- Grandparents-in-law:
- Other:

1. Birth order: First born | Only Child | Middle Child | Twin
2. Religion: Hinduism | Islam | Christainity | Zoroastriansim | Buddhism | Jainisim | Sikhism | Judaism | Other | Atheism
3. Caste: ST | SC | OBC | NT| General |
4. Profession:
5. Profession since when:
6. Household income (Per month)(Based on ****** data, give income brackets):
7. Personal income (Per month)
8. Since when have you been a ******* beneficiary/any other microfinance group
9. Have you been diagnosed with COVID-19? Yes| No
10. Have you taken the vaccine for COVID-19? Yes| No (If yes, how many doses?)
11. Place where you are giving this FGD from:

***Some group norms to be established before the FGD begins (to be conveyed to the participants):***

1. If you feel uncomfortable speaking about this topic, please mention this to me and I will take appropriate measures to ensure your comfort.
2. We want to listen to everyone’s thoughts and life stories today. So please do not interrupt when someone is speaking.
3. Please do not discourage anyone from speaking. If you do not agree with what is being spoken, please wait till the person finishes to share your thoughts.

***Instructions for FGD questions (the questionnaire will also be provided to you separately):***

***Section 1: Shared Language (to understand individual/group perception of mental health & illness)***

1. **What does mental health mean? How do you all discuss it in your community?**

***QO****: To get an understanding of what they think mental health is, and see how they respond to the term “mental health”. Further, to understand if this is discussed in their community. (What are the terms used to describe mental health; what is the meaning given to “mental health”; how is it discussed (is it hushed, discussed publicly, acknowledged, given importance?).*

*Please note that it is very important to get this concept clear as the rest of the FGD depends on this. Therefore, you may spend as much time as necessary on this question.*

***GP: Mental health*** *-- Mental health is the harmony of mind and body, dealing effectively with difficult life situations, and learning to cope with negative emotions. Just like physical health (example, when we feel pain), there is something called mental health. This indicates how we manage our emotions, and personal and social well-being. For example, when we experience sadness, does it affect the other aspects of our life such as our daily routine?*

***GP: Mental health issues*** *-- Just like physical health, mental health can also be negatively affected sometimes. This is called having mental health issues. For example, not being able to focus, feeling intense sadness, feeling a sense of deep loss upon losing a loved one, feeling excessive fear or anxiety, or not being able to manage one's anger, and so on.*

***GP: Community*** *-- Who are the people you consider to be in your social circle? It could be people in your home, neighborhood, village, district, culture, religion, and so on. A group of people you feel safe with, reach out to when you need help or spend time with during good or bad times.*

1. **How do you generally describe a mentally healthy person?**

***QO:*** *What is the understanding of a mentally healthy person? How are the symptoms perceived? Is this discussed openly? (Popular language/notion around a mentally healthy person).*

1. **How do you generally describe a mentally unwell person ?**

***QO:*** *What is the understanding of a mentally unhealthy person? How are the symptoms perceived? Is this discussed openly? (Popular language/notion around a mentally unwell person). Compared to “a mentally healthy” person, is there some add-on aspect here?*

***GP: Mentally unwell*** *-- A situation when people struggle with emotional concerns who are not able to cope with their emotions to the extent that it interferes with their everyday routine or in their relationships.*

1. **Have you observed that people who are mentally unwell are treated any differently? If yes, how?**

***QO:*** *To check for stigma. Are these people given any special/derogatory treatment? Are they integrated in the community?*

***GP:*** *How does your community perceive those who may be mentally unwell? Are their community/family/professional roles intact? Does the community try to help them? If they face any kind of special (good or bad) treatment because of their mental health related symptoms.*

1. **How do you all express negative emotions such as feeling unhappy/sad/angry/upset/scared etc.?**

***QO:*** *To establish shared language, to check for their insight with regards to their perceptions of negative feelings. In what context are these emotions expressed (private or public setting; to seek help or vent)?*

***GP: Negative emotions*** *--* *These are emotions that cause pain or discomfort, last for some time, and are sometimes related to a life event.*

1. **Do you all experience body aches or pains when you all feel distressed? Are these associated with any bodily changes?**

***QO:*** *To check for the association of physical and psychological symptoms, and level of understanding of and insight into mental health issues; to understand if they perceive a connection between mental and physical health.*

***GP: Physiological symptoms (bodily changes)*** *-- These are signs that show that something is not okay mentally. For example, physiologically speaking, high temperature (fever) may be a symptom of cold-cough, malaria, dengue, and so on. Therefore, I want to understand if you think that there are any body-related symptoms of mental health concerns.*

_____________________________________________________________________________________

***Section 2: Understanding Gender roles and existing structures (Exploring the existing systemic narrative into account)***

- 1. **Which common experiences do you all think may have an impact on the mental health of women?**

***QO:*** *Is there a larger theme on mental health issues within their community? Are there any common issues/stressors faced by the women in the village? (Focus on common experiences and not personal experiences). What kind of common experiences do they associate/ relate to which may have impacted women's mental health in their community?*

***GP: Common Experiences*** *-- These are experiences that you think not only you but also other women in your community may have had. These can be something at a large scale like shortage of necessary amenities (water, food, schooling), the pandemic; or small scale such as changes in one’s health, maternity related issues, neighbourhood, and so on.*

- 1. **What kind of work-related responsibilities affect your mental health? How?**

***QO 1:*** *As these women are entrepreneurs, are there any stressors in that aspect? Any common stressors in the business itself? Any stress coming from the family/competition regarding the business? Impact of COVID-19 on the business.*

***QO 2:*** *Does their work benefit their mental health? Being entrepreneurs, are these women at a different position (as opposed to women who are not entrepreneurs) to deal with stressors that other women from their community may be facing?*

- 1. **What kind of home related responsibilities affect your mental health? How?**

***QO:*** *Firstly, what are the gender roles that the women have to adhere to inside the house? Are they responsible solely for domestic care? stressors/responsibilities at home front may influence their mental health. Do these gender roles (inside the house) impact the mental health of the women?*

***GP:*** *What responsibilities do you have in your house? (If they are unable to give specific roles, give examples such as managing work/business, house expenses, taking care of children, taking care of elderly, and cooking). Further, how does this impact your mental health?*

____________________________________________________________________________________

***Debrief:***

Thank you for participating in this study. I would like to take a few minutes to tell you about the purpose of this study. The goal of this study is to understand the current narrative and perception around mental health amongst women in your community. This would help us gain insight into the popular language used to describe mental health related concerns, to understand how your gender influences your relation with your mental health, the kind of current interventions available in your community, and to further understand the areas of treatment gaps. Your participation today was greatly appreciated and will help in furthering our understanding of mental health in your community. We ask that you do not discuss this research with anyone else, because it could influence the study for other future participants. Would that be okay with you? Is it okay if we contact you after a week to check up on your wellbeing? Do you have any questions or concerns regarding your participation in this study?

**Supplemental Material B**

**Guidelines for in-depth interviews**

**Process:** Consent - Interview - Debrief/Closure

***General instructions (how to read this document):***

This is a guiding document to help you facilitate the interview process, and must be used only for internal processes, to understand how the interviews are to be conducted. Specifically, this document highlights the manner in which instructions and questions are to be administered to the participants such as the processes to follow if the participant needs any clarification or direction to respond to the questions. The purpose of this document is to aid a seamless interview process and avoid any interviewer bias that may influence participant responses.

You will find these terms in this document:

**“GP” = Guiding Probe -** You may use these terms while interviewing the participants. Ensure that these terms are used only to ***guide*** their responses, such that they are not deviated from the question objective. In doing so, the participants must not be primed in any manner.

**“QO” = Question Objective -** This is to help you understand what purpose the proposed question aims at fulfilling. This must ***not*** be used with the participant. At your own discretion, you may use other probes (without priming the participants) only to fulfill the QO.

The probes/meaning statements must only be used in case the participant is unable to understand the questions, is unfamiliar with the terminology used, or asks for any direct clarification. The guiding probes are in no way binding or definitive with regards to how the participant is supposed to respond. The document also lists “Question Objective” for each question with the motive to guide the interview.

***Instructions for taking Consent:***

The consent form (attached here, and will also be provided to you separately for the interview) will be read out to the participants to seek their willingness for participation in the study. The form includes the process of the interview, logistical details on how the interview will be carried out in various sub-groups, their rights in the process, potential benefits, risks involved, and final verbal agreement to be taken. Please keep the consent-taking process conversational in nature, and make sure that you begin recording the interview from this point onwards. It is important to reiterate that their participation in every step of the interview, that their participation is entirely voluntary, and that their relationship with ******* will not be impacted if they choose to stop the interview at any point.

Following is the consent form (it will also be provided to you separately):

**INFORMED CONSENT FORM**

**Introduction**:

Hi. How are you doing? My name is _____________. I am a (mental health professional) and a research assistant at Ananya Birla Foundation. Firstly, I want to thank you for taking your time out and meeting with us.

**Purpose:**

The Ananya Birla Foundation, Mumbai is doing a project on understanding mental health of women in rural Maharashtra.

Your participation in this study is voluntary and you can withdraw from the study at any point of time without giving any reason. If you have any doubts or feel uncomfortable at any point in the interview, please let me know.

Please be assured that personal information such as your name and phone number will be kept completely confidential and will be utilized only for research purposes such as coordinating with you. The information collected through such interviews will be used to write reports that would be published in a scientific journal or presented in a conference after removing your personal details. No identifying information will be mentioned.

*Do you have any questions?*

**For in person interviews**

In this interview, I will ask you (all) a few questions and your answers will be audio-recorded. The recordings will be kept safe and not shared with anyone other than the researcher.

**For video+audio (village and ******* )**

A part of this interview will be done using video call and another part on a telephone call. The audio part of this interview will be recorded. *(For those at the village: I want to point out that the ******* FO is outside the room. So if you face any technical issues, please feel free to call him).*

*Do you have any questions?*

**Benefits, Risks, and Costs**

You will be given a token of gratitude for participating in this study in the form of a 300 rupees mobile recharge voucher. *(For those who are travelling: your travel to and fro the office will be covered by us)*

During the interview, you may experience some discomfort or distress when talking about some of your experiences or those of women in your community. If this happens, please let me know and I will address your concerns to the best of my ability. If need be, we will stop the interview process. I will also guide you to an appropriate support system. After participating in this study, you may want to talk about your mental health more. We will also share some helpline numbers with you, for any emergencies regarding mental health.

I want to assure you that your decision to participate in this study will in no way affect your professional relationship with ******* .

*Any doubts/clarifications so far?*

**COVID 19 Protocols**: We are taking the necessary precautions for COVID-19 by keeping provisions for masks, hand sanitizers, and maintaining physical distancing when doing the interview in person and otherwise.

**Verbal Consent (audio recorded)**

Participant:- *“I have been informed that I am being asked to participate in a research which will explore the experiences of mental health of women in my community.*

*I understand that I have been asked to be a participant in the project. I am willing to give my time for the recorded interview. I have understood how my data will be collected and shared, and that I have the right to refuse my consent or to withdraw from the study at any point, should I like. There are no costs of withdrawing from the study”*

*Researcher: Yes, you are right.*

***Disclaimers: Any point in the interview, if the participant:***

a) **breaks down** - pause and ask if they are ok to continue, and would like a few minutes to feel better. In case they do not seem okay, please discontinue the interview and provide them with the list of mental health care resources (appended in the Debrief section).

b) **appears disoriented** - pause and check if they are feeling okay, and would like to continue with the interview process. In case they are not okay, please discontinue the interview with them, and provide them with the list of mental health care resources (appended in the Debrief section).

c) **takes long pauses when answering** - check with them after a gap of 30 seconds has elapsed by asking questions such as “What are you thinking right now?” or “What would you like to answer?” If they seem to be thinking, do not interrupt their thought process.

d) **does not seem oriented to the questions** being asked by either going off tangent or providing information irrelevant to the question - Repeat the question as is, and use probes to guide them to respond in line with the QO. Also ask “What do you think I am asking you in this question?”

***Instructions for Demographics***

Demographic details need to be taken independently for each participant. This needs to be done at the start of the study. Please ensure that FOs are not in the room while recording the interview.

**Demographic form (will also be provided to you separately):**

1) Name:

2) Age (in years):

3) Year of Birth:

4) Marital Status: Single | Married | Separated | Divorced | Widowed

5) Educational Level: Below 10th | 10th | 12th | Graduation | Post-graduation | Diploma | Vocational courses

6) Type of family (nuclear/joint):

*(Nuclear -- Living with parents and siblings if unmarried; partner if married)*

*(Joint -- Living with parents, grandparents and siblings if unmarried; living with in - laws, siblings and family if married)*

7) Number of people in your household:-

8) Number of:-

● Children (below 18 years of age):

● Children (above 18 years of age):

● Adults (above 60 years of age):

● Siblings:

● Siblings in law:

● Parents:

● Parents-in-law:

● Grandparents:

● Grandparents-in-law:

● Other:

9) Birth order: First born | Only Child | Middle Child | Twin

10) Religion: Hinduism | Islam | Christainity | Zoroastriansim | Buddhism | Jainisim | Sikhism | Judaism | Other | Atheism

11) Caste: ST | SC | OBC | NT| General |

12) Profession:

13) Profession since when:

14) Household income (Per month)(Based on ******* data, give income brackets):

15) Personal income (Per month)

16) Since when have you been a Svantantra beneficiary/any other microfinance group

17) Have you been diagnosed with COVID-19? Yes| No

18) Have you taken the vaccine for COVID-19? Yes| No (If yes, how many doses?)

19) Place where you are giving this interview from:

***Instructions for interview questions (the questionnaire will also be provided to you separately): Section 1: Shared Language (to understand individual/group perception of mental health & illness)***

**a. What is mental health according to you?**

***QO****: To get an understanding of what they think mental health is, and see how they respond to the term “mental health”.*

*Please note that it is very important to get this concept clear as the rest of the interview depends on this. Therefore, you may spend as much time as necessary on this question.*

***GP: Mental health*** *-- Mental health is the harmony of mind and body, dealing effectively with difficult life situations, and learning to cope with negative emotions. Just like physical health (example, when we feel pain), there is something called mental health. This indicates how we manage our emotions, and personal and social well-being. For example, when we experience sadness, does it affect the other aspects of our life such as our daily routine?*

***GP: Mental health issues*** *-- Just like physical health, mental health can also be negatively affected sometimes. This is called having mental health issues. For example, not being able to focus, feeling intense sadness, feeling a sense of deep loss upon losing a loved one, feeling excessive fear or anxiety, or not being able to manage one's anger, and so on.*

**b. What does community mean to you?**

***QO:*** *Who are their “in-group” (basically to set a baseline for the other questions which mention “community”). Figure out who they think is a part of their community - establish a reference group.*

***GP: Community*** *-- Who are the people you consider to be in your social circle? It could be people in your home, neighborhood, village, district, culture, religion, and so on. A group of people you feel safe with, reach out to when you need help or spend time with during good or bad times.*

**c. What does mental health mean in your community? Is mental health discussed in your community? If yes, how?**

***QO:*** *What are the terms used to describe mental health; what is the meaning given to “mental health”; how is it discussed (is it hushed, discussed publicly, acknowledged, given importance?). Also check this on a personal level.*

**d. Can you describe a mentally healthy person in your community?**

***QO:*** *What is the community’s understanding of a mentally healthy person? How are the symptoms perceived? Is this discussed openly? (Popular language/notion around a mentally healthy person).*

**e. What does being mentally unwell mean in your community?**

***QO:*** *What is the community’s understanding of a mentally unhealthy person? How are the symptoms perceived? Is this discussed openly? (Popular language/notion around a mentally unwell person). Compared to “a mentally healthy” person, is there some add-on aspect here?*

***GP: Mentally unwell*** *-- A situation when people struggle with emotional concerns who are not able to cope with their emotions to the extent that it interferes with their everyday routine or in their relationships.*

**f. What are some of the words used to describe people who might be mentally unwell?**

***QO:*** *To establish shared language/check for stigmatizing words/get insight into their vocabulary for a person who has mental health related concerns.*

**g. Can you tell me about a recent time when you were upset? Did you experience any body symptoms such as ache in your head, stomach, back or chest when you were upset? Do you think the two are connected?**

***QO:*** *To check for the association of physical and psychological symptoms, and level of understanding of and insight into mental health issues; to understand if they perceive a connection between mental and physical health.*

***GP: Physiological symptoms*** *-- These are signs that show that something is not okay mentally. For example, physiologically speaking, high temperature (fever) may be a symptom of cold-cough, malaria, dengue, and so on. Therefore, I want to understand if you think that there are any body-related symptoms of mental health concerns.*

**h. Have you observed if women in your community report similar aches or pains when they feel upset?**

***QO:*** *To check for the association of physiological and psychological symptoms...level of understanding of mental health issues; to understand if they perceive a connection between mental and physical health.*

***GP: Psychological Symptoms*** *-- These are emotional or mental symptoms that tell us that something is not okay with us. They can range from sleep difficulties, anger issues, crying, excessive worry, feeling very low in energy, not able to feel hopeful about the future, too much or too little eating, feeling insecure about self or others, having difficulty trusting others etc.*

***GP: Physiological Symptoms*** *-- These are symptoms of our body. They can include sudden weight gain or loss, changes in appetite, aches and pains in various parts of the body, feeling palpitations or perspirations, difficulties with digestion, frequent hair fall, skin issues etc.*

**i. How do women in your community express negative emotions such as feeling unhappy/sad/angry/upset/scared etc?**

***QO:*** *To establish shared language, to check for their insight with regards to their perceptions of negative feelings, and how they think other women from their community express emotions. In what context are these emotions expressed (private or public setting; to seek help or vent)?*

***GP:*** *These are emotions that cause pain or discomfort, last for some time, and are sometimes related to a life event.*

**j. Have you observed that people who are mentally unwell are treated differently? If yes, how?**

***QO:*** *To check for stigma. Are these people given any special/derogatory treatment? Are they integrated in the community?*

***GP:*** *How does your community perceive those who may be mentally unwell? Are their community/family/professional roles intact? Does the community try to help them? If they face any kind of special (good or bad) treatment because of their mental health related symptoms.*

_____________________________________________________________________________________

***Section 2: Understanding Gender roles and existing structures (Exploring the existing systemic narrative into account)***

**a. What common experiences impact the mental health of women in your community?**

***QO:*** *Is there a larger theme on mental health issues within their community? Are there any common issues/stressors faced by the women in the village? (Focus on common experiences and not personal experiences). What kind of common experiences do they associate/ relate to which may have impacted women's mental health in their community?*

***GP: Common Experiences*** *-- These are experiences that you think not only you but also other women in your community may have had. These can be something at a large scale like shortage of necessary amenities (water, food, schooling), the pandemic; or small scale such as changes in one’s health, maternity related issues, neighbourhood, and so on.*

**b. What kind of work related responsibilities affect the mental health of women in your community? How?**

***QO 1:*** *As these women are entrepreneurs, are there any stressors in that aspect? Any common stressors in the business itself? Any stress coming from the family/competition regarding the business? Impact of COVID-19 on the business.*

***QO 2:*** *Does their work benefit their mental health? Being entrepreneurs, are these women at a different position (as opposed to women who are not entrepreneurs) to deal with stressors that other women from their community may be facing?*

c. **Does having specific roles and responsibilities such as managing work/business, house expenses, taking care of children, taking care of elderly, cooking, etc. affect the mental health of women in your house? How?**

***QO:*** *Firstly, what are the gender roles that the women have to adhere to inside the house? Are they responsible solely for domestic care? stressors/responsibilities at home front may influence their mental health. Do these gender roles (inside the house) impact the mental health of the women?*

d. **Does having specific roles and responsibilities such as managing work/business, house expenses, taking care of children, taking care of elderly, cooking, etc affect the mental health of women in your community? How? (probe: for better or for worse)**

***QO:*** *Are the aforementioned stressors the same across the community (shared stressors owing to gender roles)*

_____________________________________________________________________________________

***Debrief:***

Thank you for participating in this study. I would like to take a few minutes to tell you about the purpose of this study. The goal of this study is to understand the current narrative and perception around mental health amongst women in your community. This would help us gain insight into the popular language used to describe mental health related concerns, to understand how your gender influences your relation with your mental health, the kind of current interventions available in your community, and to further understand the areas of treatment gaps. Your participation today was greatly appreciated and will help in furthering our understanding of mental health in your community. We ask that you do not discuss this research with anyone else, because it could influence the study for other future participants. Would that be okay with you? Do you have any questions or concerns regarding your participation in this study?

**FGD + Interview Questions in Marathi**

**Section 1:** Shared Language (to understand individual/group perception of mental

health & illness)

वि भाग 1: सामायि क भाषा (मानसि क आरोग्य आणि आजाराबद्दल वैयक्ति क/गट धारणा समजून

घेण्यासाठी)

a. What does mental health mean? How do you all discuss it in your community?

अ. मानसि क आरोग्य म्हणजे काय? आपण सर्व आपल्या समाजात याबद्दल काय चर्चा करता?

QO:

मानसि क आरोग्याचे वर्णन करण्यासाठी कोणते शब्द वापरले जातात ?

मानसि क आरोग्याला काय अर्थ दि ला जातो ?

मानसि क आरोग्यावर चर्चा सगळ्यांसमोर होते का ?

GP:

Mental health: मानसि क आरोग्य म्हणजे मन आणि शरीर यांचा सुसंवाद. कठीण जीवन परि स्थि ती

आणि नकारात्मक भावनांचा सामना करायला शि कणे. जसे शारीरि क आरोग्य

(म्हणजे , जेव्हा आपल्याला वेदना होतात तेव्हा/ताप येतो तेव्हा ), मानसि क

आरोग्य हि pan एक तशीचगोष्ट असते . आपण आपल्या भावना आणि स्वतःचा व समाजाचे कल्याण

कसे घडवतो हे मानसि क आरोग्यावर ठरते . उदाहरणार्थ, जेव्हा आपण दुख्खी होतो , त्याचा आपल्या

दि वसभराच्या इतर कामांवर कसा परि णाम होतो ?

Mental Health Issues: शारीरि क आरोग्य सारखाच आपल्या मानसि क आरोग्यावर कधी कधी

नकारात्मक परि णाम होतो त्यालाच मानसि क आरोग्याच्या समस्या असणं म्हणतात . मी तुम्हाला

मानसि क आरोग्याच्या समस्यांचे उदहारण देते, जसे की लक्ष्य नाही लागणे, खूप दुखी वाटणे, आपला

जवळचा माणूस गेल्यावर मनातून रि कामे वाटणे, खूप घाबरणे किंवा रागावर नि यंत्रण न आणू शकणे.

B. What does community?

तुमच्यासाठी समुदायाचा अर्थ काय?

Community: तुमच्या सामाजि क वर्तुळात तुम्ही कोणाकोणाला गणता ? त्यात तुमचे घर, परि सर,

गाव, जि ल्हा, संस्कृती, धर्म इ.लोक असू शकतात. तुम्हाला ज्यांच्या बरोबर सुरक्षित वाटतं आणि

तुम्हाला गरज असल्यास तुम्ही त्यांची मदत घेता किंवा चांगल्या किंवा वाईट वेळेत ज्या लोकांबरोबर

वेळ घालवता, त्याला सामाजि क वर्तुळ म्हणू शकता .

C.तुमच्या समाजात मानसि क आरोग्याचा अर्थ काय? तुमच्या समुदाय मध्ये मानसि क आरोग्यावर

चर्चा होते का ? जर होय, कसे?

QO:

मानसि क आरोग्याचे वर्णन करण्यासाठी कोणते शब्द वापरले जातात ?

मानसि क आरोग्याला काय अर्थ दि ला जातो ?

मानसि क आरोग्यावर चर्चा सगळ्यांसमोर होते का ?

D. Can you describe a mentally healthy person in your community?

तुम्ही तुमच्या समाजातील मानसि क आजारानं पासून नि रोगी व्यक्तीचे वर्णन करू शकता का?

QO: मानसि क आजारानं पासून नि रोगी व्यक्तीबद्दल समाजाची समज काय आहे? त्याची लक्षणे

कशी ओळखली जातात?

यावर उघड चर्चा आहे?

E.What does being mentally unwell mean in your community?

तुमच्या समाजात मानसि क आरोग्यामुळे अस्वस्थ असण्याचा अर्थ काय आहे?

QO: मानसि क आरोग्यामुळेअस्वस्थ व्यक्तीबद्दल समुदायाची समज काय आहे? त्याची लक्षणे कशी

ओळखली जातात?

यावर उघड चर्चा आहे?

GP:

Mentally unwell:अशी परि स्थि ती जेव्हा लोक भावनि क चि तं ांसह लढत असतात पण ह्या लढाईत ते

सक्षम नाही होऊ शकत .आणि ह्या गोष्टीचा त्यांच्या दि न्चार्येवर आणि वैयक्ति क समंधांवर परि णाम पडतो.

F. What are some of the words used to describe people who might be mentally unwell?

मानसि क आरोग्यामुळेअस्वस्थ असलेल्या लोकांचे वर्णन करण्यासाठी कोणते शब्द वापरले जातात ?

G. Can you tell me about a recent time when you were upset? Did you experience any body

symptoms such as ache in your head, stomach, back or chest when you were upset? Do you

think the two are connected?

ह्या काही दि वसात तुम्ही कधी नाराज होता का ? तुम्हाला नाराज असल्यामुळे काही शारीरि क अनुभव

जसे कि डोके, पोट, पाठ किंवा छाती दुखणे या सारखा काही झ्हाला का? तुम्हांला वाटते का

कि या दोन्हींचा संबंध आहे ?

GP:

Physiological changes (bodily symptoms) : ही अशी चि न्हे आहेत जी दाखवतात की एक

माणूस मानसि क आश्वासठेला सामोरी जातोय .मी तुम्हाला एक उद्धरण देते, शारीरि क आरोग्याप्रमाणे,

उच्च तापमान, सर्दी -खोकला, हे मलेरि या, डेंग्युएचे लक्षण असू शकते, तसेच मला हे जाणून घ्यायचाय कि

मानसि क आरोग्याची अस्वस्था जाणवते तेव्हा त्या बरोबर काही शरीराशी संबंधि त लक्षणे जुडलेली

असतात का?

H. Have you observed if women in your community report similar aches or pains

when they feel upset?

तुमच्या समुदायातील स्त्रि या जेव्हा नाराज होतात तेव्हा त्यांना अश्याच काही शारीरि क वेदना होतात असे

त्यांनी कधी तुम्हाला सांगि तलंय का?

GP:

Psychological Symptoms: ही भावनात्मक कि ंवा मानसि क लक्षणे आहेत जी आपल्याला ते

सांगतात आमच्याबरोबर काहीतरी ठीक नाही.काही मानसि क लक्षणे म्हणजे झोपेच्या अडचणी, रागाच्या

समस्या, रडणे, जास्त प्रमाणात काळजी, उर्जा खूप कमी वाटणे, भवि ष्याबद्दल आशावादी वाटू शकत

नाही, खूप कि ंवा खूप कमीखाणे, स्वतःबद्दल कि ंवा इतरांबद्दल असुरक्षि त वाटणे, इतरांवर वि श्वास

ठेवण्यास त्रास होणे.

Physiological Symptoms: ही आपल्या शरीराची लक्षणे आहेत. .काही शारीरि क लक्षणे

म्हणजेअचानक वजनवाढणं कि ंवा कमी होणं , भूक लागण्या मध्ये बदल, शरीराच्या वि वि ध भागात

वेदना, धडधडणे कि ंवा घाम येणे, पचनास अडचण, वारंवार केस गळणे, त्वचेच्या समस्या इ

I.How do women in your community express negative emotions such as feeling

unhappy/sad/angry/upset/scared etc?

तुमच्या समाजातील स्त्रि या नकारात्मक भावना जसे उदासीन/दु: खी/रागावलेले/अस्वस्थ/घाबरणे इ.

कशा व्यक्त करता?

GP:

Negative Emotions: या अशा भावना आहेत ज्यामुळे वेदना कि ंवा अस्वस्थता येते, काही काळ टि कते

आणि कधीकधी जीवनातील घटनेशी संबंधि त असतात.

J.Have you observed that people who are mentally unwell are treated differently?

If yes, how?

जे लोक मानसि आरोग्यामुळे अस्वस्थ आहेत त्यांना इतर लोक वेगळी वागणूक देतात असे तुम्ही पाहि ले

आहे का ? जर होय, काय वेगळी वागणूक देतात ?

QO: ह्या लोकांबरोबर काही चुकीची वागणूक केली जाते का ? त्यांना तुमच्या समाजामध्ये मानाची जागा

दि ली जाते का ?

GP: तुमचा समुदाय मानसि कदृष्ट्या अस्वस्थ असलेल्यांना कसे समजतो? त्यांचे

समुदाय/कुटुंब/व्यावसायि क भूमि का आधीसारख्या आहेत का?समाज त्यांना मदत करण्याचा प्रयत्न

करतो का?त्यांना मानसि क आरोग्याशी संबंधि त लक्षणांमुळे काही वि शेष वागणुकीला (चांगले कि ंवा

वाईट) सामोरे जावे लागत का ?

**Section 2:** Understanding Gender roles and existing structures (Exploring the existing

systemic

narrative into account)

a. What common experiences impact the mental health of women in your community?

a.सगळ्यांना येतात अशा कोणत्या अनुभवांचा महि लांच्या मानसि क आरोग्यावर परि णाम होतो?

QO- त्यांच्या समुदायामध्ये सर्वां नाच येतात अशा मानसि क आरोग्याच्या समस्या आहेत का?

गावातील महि लांना येणाऱ्या काही समस्या/तणाव समान आहेत का? (वैयक्ति क नाही

सामूहि क अनुभवांवर भर द्यावा) कुठले समान अनुभव आहेत ज्यांचा समाजातील महि लांच्या

मानसि क आरोग्यावर परि णाम झाला असेल आणि त्यांना ही ते आलेत?

GP- Common Experiences- असे अनुभव जे फक्त तुम्हालाच नाही तर सगळ्या महि लांना आले

असतील. हे मोठ्या प्रमाणाचे असू शकतात जसा कि गरजेच्या सुवि धांची कमतरता (पाणी, अन्न,

शाळा), महामारी किंवा लहान सहान अनुभव जसा तब्येतीतले बदल, मातृत्व संबंधि त समस्या,

शेजाऱ्यांबद्दल अनुभव.

b. What kind of work related responsibilities affect the mental health of women in your

community? How?

b. काम/व्यवसाया संबंधि त कोणत्या प्रकारच्या जबाबदाऱ्या तुमच्या समाजातील महि लांच्या

मानसि क आरोग्यावर परि णाम करतात? कसा?

QO1- ह्या महि ला उद्योजक आहेत म्हणून त्यामुळे काही तणाव येतो का? कामातच येणाऱ्या काही

समान समस्या आहेत का ? कामाबद्दल घरच्यांकडून/स्पर्धेतून येणारा काही ताण आहे का ?

व्यवसायावर COVID-19 चा परि णाम.

QO2- त्यांच्या कामामुळे त्यांच्या मानसि क आरोग्याला फायदा होतो का? उद्योजि का असल्याने ह्या

महि ला इतर महि लांपेक्षा( ज्या उद्योजि का नाहीत ) वेगळ्या ताणाला सामोरे जाव लागेल अशा

स्तरावर आहेत का?

c. Does having specific roles and responsibilities such as managing work/business, house

expenses, taking care of children, taking care of elderly, cooking, etc. affect the mental health

of women in your house? How?

c.काम/व्यवसाय, घरखर्च, मुलांची, मोठ्यांची काळजी घेणे, स्वयंपाक करणे अश्या वि शि ष्ट

जबाबदाऱ्यांचा तुमच्या घरातील महि लांच्या मानसि क आरोग्यावर परि णाम होतो का? कसा?

QO:महि लांनी पूर्ण करायच्या असतात कोणत्या जबाबदाऱ्या त्यांच्या घरात आहेत? त्यांच्यावर फक्त

घरगुती जबाबदाऱ्या असतात का? (घरातील)अश्या वि शि ष्ट जबाबदाऱ्यांचा महि लांच्या मानसि क

आरोग्यावर परि णाम होतो का?

d. Does having specific roles and responsibilities such as managing work/business, house

expenses, taking care of children, taking care of elderly, cooking, etc affect the mental health

of women in your community? How? (probe: for better or for worse)

d. काम/ व्यवसायाकडे लक्ष देणे, घरखर्च, मुलांची, मोठ्यांची काळजी घेण, स्वयंपाक अश्या वि शि ष्ट

जबाबदाऱ्यांचा तुमच्या समाजातील महि लांच्या मानसि क आरोग्यावर परि णाम होतो का? कसा?

(प्रोब-चांगल्या किंवा वाईट पद्धतीने)

QO:इथे सांगि तलेल्या गोष्टी ताण नि र्मा ण करतात असे समाजात सगळ्यांनाच वाटते का?

**Section 3:** Existing infrastructure and support system (prevalent mediums to deal/cope

with the symptoms/experiences)

a. What do you call people who treat mental health concerns?

मानसि क आरोग्याच्या समस्यांवर उपचार करणाऱ्या लोकांना तुम्ही काय म्हणता?

b. Whom do the women in your community go to for support/help/solution when they have

mental health concerns? (E.g. family, friends, Asha workers, etc)

तुमच्या समाजातील स्त्रि यांना मानसि क समस्या असल्या तर त्या

कोणाकडे आधार/मदत/उपायांसाठी जातात? (उदा. कुटुंब, मि त्र, आशा कामगार इ.)

QO- मानसि क आरोग्यासाठी काम करणाऱ्या लोकांबद्दल काय माहि ती आहे, अश्या समस्यांसाठी

कोणाची मदत घेतली जाते?

GP- मानसि क आरोग्याच्या समस्यां बद्दल तुम्ही कोणाजवळ बोलू शकता? तुमच्या आजूबाजूचे

कोणीही जसे कि नातेवाईक किंवा मि त्र मैत्रि णी.

c. How many times have you approached friends for support? (Follow up: how has this

helped?)

तुम्ही आधारासाठी कि ती वेळा मि त्र/मैत्रि णींकडे गेले आहात? (पाठपुरावा: यामुळे कशी मदत झाली?)

d. How has COVID 19 pandemic impacted your mental health and that of the people in your

community? (If they mention - economically - prompt - how do you think that affected them

mentally?)

कोवि ड 19 महामारीने तुमच्या आणि तुमच्या समाजातील लोकांच्या मानसि क आरोग्यावर कसा

परि णाम झाला आहे? (जर त्यांनी उल्लेख केला - आर्थि कदृष्ट्या - सुचवा- तुम्हाला असे कसे वाटते की

त्याचा त्यांच्यावर मानसि क परि णाम झाला?)

e. How has your community coped with COVID-19 as a whole? (Probe: support systems)

तुमच्या समुदायाने कोवि ड -19 चा सामना कसा केला? (प्रोब: कोणी आधार दि ला किंवा मदत केली )

QO- COVID 19 मध्ये आलेल्या मानसि क ताणाचा सामना करणे, समुदायातून मि ळणार आधार?

महामारीत येणाऱ्या ताण किंवा वाईट भावनांशी सामना करायला कुठल्या गोष्टींची मदत झाली.

GP- support systems- म्हणजे अशा जागा लोकं आणि अनुभव जे आपल्याला अवघड काळात

आधार देतात. हे लोकं तुमच्या समुदायात किंवा बाहेरील कोणी असेल, कोणी पुढारी, कुठलेही गट,

उपक्रम असे काहीही.

f. Have you observed that women in your community seek help for mental health concerns?

If yes, from whom and where?

तुमच्या समाजातील स्त्रि या मानसि क आरोग्याच्या समस्यांसाठी मदत घेतात असे

तुम्ही पाहि ले आहे का? जर होय, कोणाकडून आणि कुठून?

QO- मानसि क आरोग्याच्या समस्यांसाठी मदत घेण्या बद्दल समज; कोणत्या प्रकारची मदत घेतली

जाते? कुठल्या प्रकारच्या तज्ज्ञाची मदत घेतली जाते?

GP- Alternate healing- साधारण डॉक्टर वि ज्ञाना प्रमाणेपेशंटला देतात त्या उपचारांपेक्षा हे वेगळे

असतात. जसं कि रेकी, acupuncture (शरीरात सूया टोचून रोग बरा करणे), मसाज, एखाद्या

अध्यात्मि क गुरु/ साधू/ आयुर्वेदि क डॉक्टरकडे जाणे.

g. Under what conditions do women in your community seek help (from their previous

responses; refer: 3e)?

तुमच्या समाजातील स्त्रि या कोणत्या परि स्थि तीत मदत घेतात (त्यांच्या आधीच्या उत्तरांमधून

पहा: 3e)?

QO- मानसि क आरोग्याबद्दल मदत घेण्यासाठी काय नि कष असतात ? कोणत्या

परि स्थि ती/चि न्हे/लक्षणे असतील तर मानसि क आरोग्यासाठी मदत घेतली जाते.

GP- मानसि क आरोग्यासाठी महि ला इतर कोणाची मदत कि ती त्रास होत असेल तर घेतात?

h. Do you have people who visit your community to provide support/ help/ solutions to treat

mental health concerns? If yes, can you describe their role?

मानसि क आरोग्याच्या समस्यांवर आधार/मदत/ उपचार करण्यासाठी तुमच्या समुदायाला भेट देणारे

लोक आहेत का? असे लोक येत असतील तर त्यांची काय भूमि का असते?

GP- Asha workers- आशा स्वयंसेवक गावातील आरोग्य वि षयक समस्यांसाठी काम करतात

(उदाहरणार्थ)

i. What are some practices women in your community engage in to take care of their mental

health? (Probe - checking for self-care: yoga, meditation, AYUSH guidelines etc.)

मानसि क आरोग्याची काळजी घेण्यासाठी आपल्या समाजातील स्त्रि या कोणत्या पद्धतींचा उपयोग

करतात? (प्रोब - स्वत: ची काळजी घेणे: योग, ध्यान, आयुष मार्गदर्शक तत्त्वे इ.)

GP- self-care- आपण स्वतःची काळजी घेण्यासाठी करतो त्या गोष्टी. ह्याचा अर्थ आराम करणे,

मोकळ्या वेळेत आपल्या आवडीच्या गोष्टी करणे, आवडीच्या लोकांबरोबर वेळ घालवणे, पुरेशी झोप

घेणे , योगा, ध्यान करणे, आयुष मार्गदर्शक तत्त्वे (म्हणजेथोडक्यात आयुर्वेद, योग, होमि ओपॅथी

इत्यादी) पाळणे.

j. Under what conditions have you observed that mental health concerns become better or

worse?

कोणत्या परि स्थि तीत मानसि क आरोग्याच्या तक्रारी बऱ्या होताना किंवा ढासळताना तुम्ही पाहि ले

आहे?

QO- आधी केलेल्या उपायांचा फायदा झाला का, त्यांना अजून कुठला उपाय काम करेल असं वाटतं?

त्यांच्या मते कोणत्या गोष्टींनी मानसि क आरोग्य ढासळते ? कोणत्या गोष्टींमुळे मानसि क आरोग्य

चांगले होते?

**Section 4:** Potential Interventions (Feasibility)

a. Would women in your community feel comfortable if an outsider/non community person

speaks to them about mental health? If not, who would they prefer?

एखादाअनोळखी /समुदाया बाहेरचा माणूस मानसि क आरोग्याबद्दल बोलला तर तुमच्या

समाजातील स्त्रि यांना बरं वाटेल का? नाही, तर त्या कोणाला पसंत करतील?

b. What sort of help is more likely to be taken up by women from your community? For

example, talking to a psychiatrist, counselor, support group, group therapy, tele-health?

तुमच्या समाजातील स्त्रि यांनी कशा प्रकारची मदत घेण्याची शक्यता जास्त

आहे? उदाहरणार्थ, मनोवि कारतज्ज्ञां म्हणजेच मानसि क स्वास्थ डॉक्टरशी बोलणे, समुपदेशक किंवा

सल्लागार , मदत गट, सामूहि क उपचार, टेलेफोनवरून डॉक्टरशी संभाषण ?

c. What kind of solution do you think would help improve the mental health of women in

your community?

तुमच्या समाजातील स्त्रि यांचे मानसि क आरोग्य सुधारण्यासाठी कोणत्या उपायांची मदत होईल

असे तुम्हाला वाटते?

d. Is there anything specific we need to keep in mind when providing interventions? (time of

the year, absence/presence of family members, group/ individual).

मानसि क स्वास्थासंदर्भा त जर आम्हाला काही मदत पोहचवायची असेल तर तुमच्या मते आम्ही काही

ठरावि क गोष्टी लक्षात घ्यायला हव्या का? (वेळ, कुटुंबातील सदस्यांची अनुपस्थि ती/

उपस्थि ती,सामूहि क / वैयक्ति क).

**Section 5: Feedback** (Recommendations for future interviewing)

वि भाग 5: अभि प्राय (भवि ष्यातील मुलाखतीसाठी शि फारसी)

a. How was your experience with the interview? Would it be different if the interview is

conducted face-to-face/on call? Would you prefer a different medium of communication

for the

interview (example, only video, only audio, video+audio)?

मुलाखतीचा तुमचा अनुभव कसा होता? जर मुलाखत समोरासमोर/कॉलवर घेतली गेली असती तर

अनुभव वेगळा असता का ? तुम्ही मुलाखतीसाठी संवादाचे वेगळे माध्यम पसंत कराल?

B. How did you feel while answering the questions during the interview?

B. मुलाखतीदरम्यान प्रश्नांची उत्तरे देताना तुम्हाला कसे वाटले?

C. Did you understand what the interviewer meant? Did you feel that it would have

been more comfortable if it was done differently? If yes, how?

Did you understand what the interviewer meant? Did you feel that it would have been

more comfortable if it was done differently? If yes, how?

c मुलाखतकाराला काय म्हणायचे आहे ते समजले का? जर हे प्रश्न वेगळ्या पद्धतीने केले गेले

असते तर जास्त योग्य आणि समजण्यासाठी सोप्पे झाले असते, तुम्हाला वाटते का ? जर होय, कसे?

d. What according to you is the best place to conduct this interview for women in your

community?

d तुमच्या मते तुमच्या समाजातील महि लांसाठी ही मुलाखत देण्यासाठी सर्वो त्तम ठि काण कोणते

आहे?

e. How was your experience travelling to the Svantantra office for an interview?

e मुलाखतीसाठी स्वंतंत्र कार्या लयात प्रवास करताना तुमचा अनुभव कसा होता?

f. Did you learn anything new through this interaction?

f या संवादातून तुम्ही काही नवीन शि कलात का?

**Debrief:** Thank you for participating in this study. I would like to take a few minutes to

tell you about the purpose of this study. The goal of this study is to understand the

current narrative and perception around mental health amongst women in your

community. This would help us gain insight into the popular language used to describe

mental health related concerns, to understand how your gender influences your relation

with your mental health, the kind of current interventions available in your community,

and to further understand the areas of treatment gaps. Your participation today was

greatly appreciated and will help in furthering our understanding of mental health in

your community. We ask that you do not discuss this research with anyone else, because

it could influence the study for other future participants. Would that be okay with you?

Do you have any questions or concerns regarding your participation in this study

तुम्ही या अभ्यासात सहभागी झाल्याबद्दल धन्यवाद. आता या अभ्यासाबाबतीत तुम्हाला काही

माहि ती देणार आहे.

तुमच्या बोली भाषेतून मानसि क आरोग्या संबंधी तमु च्या चि तं ा, तमु चं स्त्री असणं तमु च्या मानसि क

आरोग्यवर काय परि णाम करत, तसच तुमच्या आजूबाजूला किंवा समाजात कोणत्या उपचार पद्धती

उपलब्ध आहे आणि त्यातल्या त्रुटी/कमतरता आम्हाला समजून घेता आल्या. आज आमच्या या

अभ्यासात/प्रकल्पात सहभागी झाल्याबद्दल आम्हाला तुम्हा सर्वां चं वि शेष कौतुक आणि आभार.

यामुळे तुमच्या समाजातल्या मानसि क आरोग्या वि षयी आम्हाला अधि क माहि ती मि ळाली. फक्त या

संशोधना/अभ्यासा बद्दल इतरांशी चर्चा न करण्याची आम्ही तुम्हाला वि शेष वि नंती करतो. आम्ही

इतर महि लांशी ही याप्रकारे संवाद साधणार आहे, तर तुम्ही या अभ्यासावि षयी त्यांच्याशी काहीही

चर्चा नाहीतर त्याच्याबरोबर होणाऱ्या संवादावर, अभ्यासावर त्याचा परि णाम होऊ शकतो. तुम्हा

सर्वां ना समजलं? तुमच्या पैकी कुणाला तुमच्या सहभागा बद्दल काही शंका, प्रश्न आहेत का?

**Shortened Consent form**

नमस्कार . तुम्ही कसे आहात? माझं नावं आहे _____________. मी लोकांच्या मानसि क

आरोग्या साठी काम करते आणि

सध्या अनन्या बि र्ला फाउंडेशनमध्ये संशोधन करत आहे . सर्वप्रथम, या भेटीसाठी वेळ काढल्याबद्दल मी

तुमचे आभार मानू इच्छि ते.मुंबईतील अनन्या बि र्ला फाउंडेशन, हे ग्रामीण महाराष्ट्रातील महि लांचे

मानसि क आरोग्य समजून घेण्यासाठी एक प्रकल्प करत आहे.

ह्या अभ्यासात तुम्ही तुमच्या इच्छे प्रमाणे भाग घेऊ शकता आणि जर तुम्हाला कोणत्याही वेळी

अस वाटलं कि तुम्हाला ह्या अभ्यासात पुढे सहभाग नाही घ्यायचाय तर तुम्ही कोणतेही कारण न देता

अभ्यासातून माघार घेऊ शकता. तुम्हाला ह्या मुलाखतीत कोणत्याही वेळी कसलीही शंका असेल कि ंवा

कसलीही माहि ती हवी असेल तर तुम्ही मला लगेच वि चारू शकता. आम्ही तूमची थोडी माहि ती लि हून

घेऊ पण ती माहि ती आणि आपण आज ज्याही गोष्टी बोलू त्याची रेकॉर्डिं ग आम्ही सांभाळून ठेऊ आणि

संशोधन उद्देशासाठीच ती माहि ती वापरू.

तुम्हाला काही प्रश्न आहेत का?

**Benefits, Risks and Costs.**

गटचर्चे च्या वेळी तुम्हाला तुमचे अनुभव सांगताना किंवा तुमच्या समाजातील स्त्रि यांचे अनुभव

सांगताना थोडा अस्वस्थ वाटलं किंवा काही त्रास झ्हाला तर तुम्ही मला लगेच सांगा, आपण हि चर्चा

गरज वाटल्यास थांबवूआणि मी माझ्या क्षमतेनुसार तुमच्या चि तं ा दरू करण्याचा प्रयत्न करेन. तुम्हाला

आपल्या भेटी नंतर तुमच्या मानसि क आरोग्या बद्दल बोलावसं वाटेल कि ंवा तुम्हाला काही त्याबाबतीत

प्रश्ण वि चारायचे असतील म्हणून आम्ही तुमच्यासोबत काही फोन क्रमांकही शेअर करू, तुम्ही ति कडे

फोन करून माहि ती आणि सल्ला घेऊ शकता.

मी तुम्हाला आश्वासन देऊ इच्छि ते की या अभ्यासात सहभागी होण्याच्या तुमच्या नि र्णयाचा तुमच्या

स्वंतत्राशी असलेल्या व्यावसायि क संबंधावर कोणताही परि णाम होणार नाही

आतापर्यंत काही शंकाआहे का ? कि ंवा कसलेही स्पष्टीकरण हवे आहे का?

आम्ही ह्या भेटीत कोवि द १९ चालू अस्तास , नीट काळजी घेतोय. मास्क, सानि टीझर आणि शारीरि क

अंतर हे सर्व नि यम नीट पाळतोय.

**Simplified Verbal Consent:**

सहभागी:

माझ्या समाजातील महि लांच्या मानसि क आरोग्याचे अनुभव समजावून घेणाऱ्या एका संशोधनात

मला सहभागी होण्यास सांगि तले जाणार आहे आणि ह्या बद्दल मला सगळी माहि ती दि ली गेली आहे.

मी इकडे जे हि बोलेन त्याची रेकॉर्डिं ग होईल आणि ती रेकॉर्डिं ग सांभाळून ठेवली जाईल आणि त्याचा

वापर फक्त अभ्यासासाठी केला जाईल हे हि मला समजलंय. मला हे हि कळलय कि मी कोणत्याही

वेळी माझ्या इच्छे नुसार ह्या अभ्यासातून मगर घेऊ शकते

**Supplemental Material C**

Behaviour Checklist

| **To be rated by interviewers, while the interview is being conducted** |
| --- |
|  |
| **Please rate from 1 (least) to 5 (most), the extent to which you observed the participant to:** |
| Hesitate when answering any questions |
| Hesitate while answering about mental health |
| Refused to talk about mental health |
| Use filler sentences at any time |
| Use filler sentences when talking about mental health |
| Need probing when answering any questions |
| Need probing when answering questions on mental health |
| Engage in tangential conversation |
| Try and change the topic |
| Displayed closed non-verbal behaviours |
| Dispayed open non-verbal behaviours |
| Rephrase their answers post probing (positive) |
| Rephrase their answers post clarification (positive) |
| Rephrase their answers post probing (negative) |
| Rephrase their answers post clarification (negative) |
| Counter question the interviewer (example, what do they think about mental health) |
| Notes (mention any specific incidents here) |

**Supplemental Material D**

**Distress Protocol**

*The protocol for managing distress in the context of a research focus group /interview*

This protocol ensures that research participant’s wellbeing is protected. This is a 10 step protocol. The steps outlined below are for participants' benefit in case they feel distressed while being interviewed.

*If a participant indicates that they are experiencing emotional distress or exhibit behaviours suggestive that the discussion/interview is too stressful such as uncontrolled crying, shaking etc., the researcher will:*

1. Pause the discussion/interview.

2. Ask the participant if they would like to take a break and if they wish for the audio-recorder to be switched off (incase of online interview).

3. If the participant is doing better, resume with the interview.

4. If the participant continues to show signs of distress, the researcher will withdraw her from the discussion and accompany her to a quiet area (in FGD; so the researcher can assess their mental status directly) and assess mental status by asking the following questions: • *Tell me the thoughts you are having right now*

• *Tell me what you are feeling right now*

• *Do you feel safe?*

5. The researcher will then help her regulate her emotions by using various techniques from the emotion regulation techniques worksheet (any one or a combination of those can be used based on the setting and availability of resources around).

6. If the participant is doing better, resume with the interview.

7. If the participant is unable to carry on, terminate the interview. In case they choose to withdraw, talk to them about a plan that might help them calm down once they reach home in terms of activity mapping for the rest of the day.

8. In case the participant is unduly distressed, the researcher will remain with the participant (incase of FGD, one of the researchers will stay with the participant until they have done at least one activity from the emotion regulation skills sheet. In case of any perceived harm to the participants physical safety, the researcher will take details of an emergency contact {someone who the participant trusts} and with the help of FO reach out to the emergency contact).

9. The researcher will, with the participants’ consent, gain permission to call them later in the day or the following day to ensure they are no longer distressed.

10. The researcher will provide contact details of useful numbers and support groups to the participant if they require them.

*Note:* The numbers in the flowchart correspond to the numbers in the aforementioned distress protocol

**Tabular format**

| Distress | If a participant indicates that they are experiencing emotional distress or exhibit behaviours suggestive that the discussion/interview is too stressful such as uncontrolled crying, shaking etc., |
| --- | --- |
| Stage 1  Response | 1. Pause the interview/discussion  2. Ask the participant if they would like to take a break and if they wish for the audio recorder to be switched off (incase of online interview). |

| Review | If the participant is doing better, resume with the interview.  If the participant is unable to carry on, go to stage 2 |
| --- | --- |
| Stage 2  Response | If the participant continues to show signs of distress, the researcher will withdraw her from the discussion and accompany her to a quiet area (in FGD; so the researcher can assess their mental status directly) and assess mental status by asking the following questions: • Tell me the thoughts you are having right now  • Tell me what you are feeling right now  • Do you feel safe?  The researcher will then help her regulate her emotions by using various techniques from the emotion regulation techniques worksheet (any one or a combination of those can be used based on the setting and availability of resources around). |
| Review | If the participant is doing better, resume with the interview (reiterating the voluntary nature of participation).  If the participant is unable to carry on, go to stage 3 |
| *Stage 3*  *response* | If the participant is unable to carry on, terminate the interview. In case they choose to withdraw, talk to them about a plan that might help them calm down once they reach home in terms of activity mapping for the rest of the day. |
| *Review* | Once the participant has calmed down, they can leave  In case the participant is unduly distressed, one of the researchers will remain with the participant (incase of FGD one of the researchers will stay with the participant until they have done atleast one activity from the emotion regulation skills sheet. In case of any perceived harm to the participants physical safety, the researcher will take details of an emergency contact {someone who the participant trusts} and with the help of FO reach out to the emergency contact). |
| *Follow up* | The researcher will, with the participants’ consent, gain permission to call them later in the day or the following day to ensure they are no longer distressed.  The researcher will provide contact details of useful numbers and support groups to the participant if they require them. |

**Distress Protocol**

*The protocol for managing distress in the context of a research focus group /interview*

This protocol ensures that research participant’s wellbeing is protected. This is a 10 step protocol. The steps outlined below are for participants' benefit in case they feel distressed while being interviewed.

*If a participant indicates that they are experiencing emotional distress or exhibit behaviours suggestive that the discussion/interview is too stressful such as uncontrolled crying, shaking etc., the researcher will:*

1. Pause the discussion/interview.

2. Ask the participant if they would like to take a break and if they wish for the audio-recorder to be switched off (incase of online interview).

3. If the participant is doing better, resume with the interview.

4. If the participant continues to show signs of distress, the researcher will withdraw her from the discussion and accompany her to a quiet area (in FGD; so the researcher can assess their mental status directly) and assess mental status by asking the following questions: • *Tell me the thoughts you are having right now*

• *Tell me what you are feeling right now*

• *Do you feel safe?*

5. The researcher will then help her regulate her emotions by using various techniques from the emotion regulation techniques worksheet (any one or a combination of those can be used based on the setting and availability of resources around).

6. If the participant is doing better, resume with the interview.

7. If the participant is unable to carry on, terminate the interview. In case they choose to withdraw, talk to them about a plan that might help them calm down once they reach home in terms of activity mapping for the rest of the day.

8. In case the participant is unduly distressed, the researcher will remain with the participant (incase of FGD, one of the researchers will stay with the participant until they have done at least one activity from the emotion regulation skills sheet. In case of any perceived harm to the participants physical safety, the researcher will take details of an emergency contact {someone who the participant trusts} and with the help of FO reach out to the emergency contact).

9. The researcher will, with the participants’ consent, gain permission to call them later in the day or the following day to ensure they are no longer distressed.

10. The researcher will provide contact details of useful numbers and support groups to the participant if they require them.

*Note:* The numbers in the flowchart correspond to the numbers in the aforementioned distress protocol

**Tabular format**

| Distress | If a participant indicates that they are experiencing emotional distress or exhibit behaviours suggestive that the discussion/interview is too stressful such as uncontrolled crying, shaking etc., |
| --- | --- |
| Stage 1  Response | 1. Pause the interview/discussion  2. Ask the participant if they would like to take a break and if they wish for the audio recorder to be switched off (incase of online interview). |

| Review | If the participant is doing better, resume with the interview.  If the participant is unable to carry on, go to stage 2 |
| --- | --- |
| Stage 2  Response | If the participant continues to show signs of distress, the researcher will withdraw her from the discussion and accompany her to a quiet area (in FGD; so the researcher can assess their mental status directly) and assess mental status by asking the following questions: • Tell me the thoughts you are having right now  • Tell me what you are feeling right now  • Do you feel safe?  The researcher will then help her regulate her emotions by using various techniques from the emotion regulation techniques worksheet (any one or a combination of those can be used based on the setting and availability of resources around). |
| Review | If the participant is doing better, resume with the interview (reiterating the voluntary nature of participation).  If the participant is unable to carry on, go to stage 3 |
| *Stage 3*  *response* | If the participant is unable to carry on, terminate the interview. In case they choose to withdraw, talk to them about a plan that might help them calm down once they reach home in terms of activity mapping for the rest of the day. |
| *Review* | Once the participant has calmed down, they can leave  In case the participant is unduly distressed, one of the researchers will remain with the participant (incase of FGD one of the researchers will stay with the participant until they have done atleast one activity from the emotion regulation skills sheet. In case of any perceived harm to the participants physical safety, the researcher will take details of an emergency contact {someone who the participant trusts} and with the help of FO reach out to the emergency contact). |
| *Follow up* | The researcher will, with the participants’ consent, gain permission to call them later in the day or the following day to ensure they are no longer distressed.  The researcher will provide contact details of useful numbers and support groups to the participant if they require them. |

**Supplemental Material E**

**Emotion Regulation Techniques**

***(Step 5 of the ‘Distress Protocol’)***

*Use any one or a combination of these based on the availability of resources and the participant receptivity.*

**Grounding Techniques**

**5-4-3-2-1 Technique**

**What are 5 things you can see?** Look around the room carefully and please tell me 5 objects that you can see around you. Or tell me 5 (name any colour) objects that you see around you.

**What are 4 things you can feel?** Notice and tell me the sensation of clothing on your body, the sun on your skin, or the feeling of the chair you are sitting in. Pick up an object and examine its weight, texture, and other physical qualities.

**What are 3 things you can hear?** Pay special attention to the sounds your mind has tuned out, such as a ticking clock, distant traffic, or trees blowing in the wind.

**What are 2 things you can smell?** Try to notice smells in the air around you, like an air freshener or a perfume (researchers can carry with them a scented candle or a perfume).

**What is 1 thing you can taste?** (Researchers can carry gum, candy, or small snacks for this step). Pop one in your mouth and focus your attention closely on the flavors. For those who are doing it online, ask them to imagine a half cut lemon on their tongue or imagine the last thing that you ate.

**Body Awareness technique**

The body awareness technique will bring you into the here-and-now by directing your focus to the sensations in the body. Pay special attention to the physical sensations created by each step.

1. Take 5 long, deep breaths through your nose, and exhale through your mouth. 2. Stomp your feet on the ground several times. Pay attention to the sensations in your feet

and legs as you make contact with the ground.

3. Clench your hands into fists, then release the tension. Repeat this 10 times. 4. Press your palms together. Press them harder and hold this pose for 15 seconds. Pay

attention to the feeling of tension in your hands and arms.

5. Rub your palms together. Notice and sound and the feeling of warmth. 6. Reach your hands over your head like you’re trying to reach the sky. Stretch like this for 5 seconds. Bring your arms down and let them relax at your sides.

7. Take 5 more deep breaths and notice the feeling of calm in your body.

**Soothing Technique**

1. Say kind words to yourself, as if you were talking to a friend or small child – for example, “I’ll get through this”.

2. Think of favorites. For e.g. think of your favorite color, animal, season, food, time of day, TV show.

3. Imagine people you care about such as your child or someone you really love. 4. Remember the words to an inspiring song, quotation, or poem that makes you feel better (e.g., serenity prayer).

5. Say a statement like “I can handle this,” “This feeling will pass.” 6. Think of things you are looking forward to in the next week – time with a friend, some activity that you are going to do.

**Supplemental Material F**

**Resource list of Mental Health organizations/helplines**

| **Orgn/Helpline** | **About** | **Number** | **Email** | **Website** |
| --- | --- | --- | --- | --- |
| i-call | Free Telephone & Email based Counseling Services  Monday, Tuesday, Wednesday, Thursday, Friday, Saturday @ 10:00AM - 8:00 PM | 022-25521111  *Owing to the lockdown across India due to Covid-19 outbreak, this helpline may be operating at lower capacity on:*  *+91 8369799513* | icall@tiss.edu | <https://icallhelpline.org/> |
| Samaritans Mumbai | A helpline providing emotional support for those who are stressed, distressed, depressed, or suicidal  Sunday, Monday, Tuesday, Wednesday, Thursday, Friday, Saturday @ 5:00 PM - 8:00 PM | +91 8422984528  *Owing to the lockdown across India due to Covid-19 outbreak, this helpline may be operating at lower capacity on:*  *+91 8422984530* | talk2samaritans@gmail.com | <https://samaritansmumbai.org/> |
| sumunum connect | A free multilingual mental health & wellbeing support line. Offering mental health crisis interventions, emotional support & handholding services.  Sunday, Monday, Tuesday, Wednesday, Thursday, Friday, Saturday @  11:00 AM - 11:00 PM | 1800-123-786868 | connect@sumunum.com | <https://www.sumunum.com/sumunum-connect?utm> |
| Mann Talks | The Mann Talks Helpline offers free and confidential emotional and psychological support through a team of trained mental health professionals  Sunday, Monday, Tuesday, Wednesday, Thursday, Friday, Saturday @ 09:00 AM - 6:00 PM | +91 8686139139 | - | <https://www.manntalks.org/> |
| Parivarthan | Parivarthan Counselling Helpline (PCH), is a free phone counselling service operated by a team of 15 trained, certified and multilingual counsellors  Monday, Tuesday, Wednesday, Thursday, Friday, @ 1:00 PM - 10:00 PM | +91 7676602602 | parivarthanblr@gmail.com | <https://parivarthan.org/> |
| Aasra | 24 *7 helpline for emotional crisis situations, mental illness issues, and suicidal ideation. | Hitguj help number  +91 022 24131212  Mumbai | [aasrahelpline@yahoo.com](mailto:aasrahelpline@yahoo.com) | <http://www.aasra.info/> |
| Mpower helpline | In order to deal with the mental health concerns arising out of the pandemic and subsequent lockdown, the BMC and Mpower 1 on 1, have started a toll-free, 24*7 helpline. | 1800 120 820050 |  | <https://mpowerminds.com/oneonone> |
| Kiran | 24/7 toll-free helpline provides support to people facing anxiety, stress, depression, suicidal thoughts and other mental health concerns. | 1800 599 0019 | - | - |
| Cooj Mental Health Foundation | Offers emotional support to those in emotional distress or feeling suicidal | 08322252525 | YouMatterByCooj@gmail.com | - |
| Sangath COVID-19 Well-being Center | Sangath’s tele-counselling services offer free phone-based counselling for frontline workers and young people in need of psychological support.  10AM-4PM, Daily | 9372048501 |  | <https://sangath.in/covid19/> |

**Supplemental Material G**

| **Global theme (linked with RQ)** | **Global theme as a question** | **Organizing theme** | **Organizing theme as a response** |  |
| --- | --- | --- | --- | --- |
| **Language** | **How do people talk about mental health in rural Maharashtra?** | **Personal meaning** | **Attributing personal meaning to mental health** |  |
|  |  | **Community's meaning** | **Attributing community norms to mental health** |  |
| **Stigma** | **What is the extent of stigma regarding mental health in rural Maharashtra?** | **Self-stigma** | **Existence of intra-personal stigma regarding mental health in rural Maharshtra** |  |
|  |  | **Public stigma** | **Existence of inter-personal stigma regarding mental health in rural Maharashtra** |  |
| **Social identity** | **How does one's social identity impact their mental health in rural Maharashtra?** | **Oppression** | **Physical harm (bullying, abuse) or emotional harm (emotional hurt, mental health issues) on account of belonging to social groups based on caste, gender, age, religion, and so on** |  |
|  |  | **Discrimination** | **Wrongful treatment on account of belonging to social groups based on caste, gender, age, religion, and so on** |  |
|  |  | **Gender roles and responsibilities** | **Perceived gender roles and responsibility** |  |
| **Presentation of mental health issues** | **What are the common mental health stressors amongst women in rural Maharashtra?** | **Antecedents** | **Mental health issues have varying causes in rural Maharashtra / The 3 most prominent triggers for mental health related issues for women in rural maharashtra are __ __ __** |  |
|  | **How are mental health issues usually presented by women in rural Maharashtra?** | **Mental health symptoms** | **Notable symptoms or presentations of mental health concerns** |  |
|  | **How are physical health issues usually presented by women in rural Maharashtra?** | **Physical health symptoms** | **Notable symptoms or presentations of physical health concerns** |  |
|  | **How adept are women in rural Maharashtra in percieving their own mental health issues?** | **Personal perceived concept** | **Self-perception and expression of mental health concerns** |  |

**Supplemental Material H**

*Coding procedure*

| **Steps** | **Description** |
| --- | --- |
| 1.      Interview | 72 interviews conducted in 6 administrative districts |
| 2.      Transcription | Recordings translated and transcribed from 32 interviews |
| 3.      Coding | Transcribed files coded based on the codebook |
| 4.      Inter - rater reliability | IRR assessed in 2 pairs using Kappa coefficient |
| 5.      Discrepancies | Discrepancies in IRR addressed between coders and other subject matter experts |
| 6.  Streamlining coding | Step 4 and 5 repeated till IRR discrepancies were resolved. |

**Supplemental Material I**

*Extract 1*: *“Mental health means… If there are any problems, it could lead to mental health issues. It affects mentally”*

*Extract 2: “So some get angry.” and “Ummm… (Long pause) irritation, madness or getting angry*”.

*Extract 3: “Mental health means our body’s… ummm… it is all part of our body right? Mental health and everything?”* and *“Mental means body’s internal thing.. that’s known as mental health”.*

*Extract 4: “Only if someone passes away in our village then I get scared and my heart beats faster and therefore, I don’t go anywhere and my husband insists that I should not leave the house because I get easily scared if I hear something like this. My blood pressure drops”*

*Extract 5: “It may happen because of tension. If someone has any tension or if there’s a fight at home, then these things may happen. That’s how we get tense*”

*Extract 6: "That’s right. If a woman is not able to conceive, she gets tensed, right? That she is not able to give birth. Everyone is able to, but she is not. And when a lot of days have passed and when women come together then women discuss and say things like "it's not too late, seek treatment, seek remedies, try other options etc".*

*Extract 7:* “*There are financial problems. And we are almost the same age.. so she must be in her forties. And as her husband passed away so early, now there are many challenges” and “Yes. If any woman doesn’t bring any money for repaying instalments then we get tense. If they don’t get money to repay etc.*”

*Extract 8:* “*Their work does not complete and if someone is an addict…who consumes alcohol, then such women may have tension and it can also affect their health. And if someone is not an addict, then nothing goes wrong. Their families are happy.*” and “*Some are tense because of addiction related problems. Everyone has some or the other problem, right*?” are a few excerpts from this basic theme.

*Extract 9*: “*I think this will also have an effect on mental health, madam. They experience head-ache. They are in pain. So some women face a lot of problems at home*.”

*Extract 10: “Mental health meaning it can be quite hectic.I too used to go to work and I recently quit one and half years ago during Covid times.. and by hectic I mean one has to get up early, do all the chores, attend to kids and their needs and after taking care of everything leave home it is physically very hectic and after returning home once again take care of everything, children etc..”*

*Extract 11:* “*Yes, ma’am. They go to the farm and then it takes a toll on their health. There’s a difference between women who work at home and those who work on farms. Ma’am, we don’t feel anything. It is our business, so we have to do it. Even if you feel something, you have to do it somehow to earn your bread”.*

*Extract 12*: “*No. Nothing like that but women have to go to work. They are just worried that.. I mean they have to go. Even if they don’t go.. their condition is such that they have to go to work. There’s no other option. They are worried about that but women can adjust. Nothing else”.*

*Extract 13: “It happens to an extent to some women. Husbands of some women have an addiction and so all the responsibilities fall on women’s shoulders. So it's going to affect their mental health, right? They get tense. Or they may get tense if the mister comes home or goes out. Then they vent out on their children. So any woman whose husband is an addict may face these types of problems''.*

*Extract 14: “Their husbands are alcoholic. They don’t go to work. Then that woman has so many responsibilities, they have to take care of their children, she goes berserk. She has no sense of what she is doing”.*

*Extract 15: “No, I mean I could not speak properly but what can I do.. I mean I am not very literate. I am a simple woman. I don't have much interest in other worldly things so I answered whatever I could”“Oh.. is it.. I don’t understand it, ma’am. I am not educated, that’s why” “I feel that I may have made some mistakes. I could not answer properly”.*

*Extract 16: “If someone is suffering from critical diseases such as TB or Cancer, then people in their surrounding might be treating them a little differently…”*
