# Supplementary material for: Perspectives and presentation of mental health among women from rural Maharashtra (India): A qualitative study
Source: Glob Ment Health (Camb). 2024 Mar 6;11:e40. doi: 10.1017/gmh.2024.28 (PMC11016358; doi:10.1017/gmh.2024.28)
Supplement: Peer review history

# Editor report: Recommendation: Perspectives and presentation of mental health among women from rural Maharashtra (India): A qualitative study — R0/PR2

**DOI:** 10.1017/gmh.2024.28.pr2

**Affiliations:** The George Institute for Global Health India, India

Pl address the concerns raised by the reviewers.

# Editor report: Decision: Perspectives and presentation of mental health among women from rural Maharashtra (India): A qualitative study — R0/PR3

**DOI:** 10.1017/gmh.2024.28.pr3

**Affiliations:** Johns Hopkins University Bloomberg School of Public Health, United States

No accompanying comment.

# Editor report: Recommendation: Perspectives and presentation of mental health among women from rural Maharashtra (India): A qualitative study — R1/PR5

**DOI:** 10.1017/gmh.2024.28.pr5

**Affiliations:** The George Institute for Global Health India, India

No accompanying comment.

# Editor report: Decision: Perspectives and presentation of mental health among women from rural Maharashtra (India): A qualitative study — R1/PR6

**DOI:** 10.1017/gmh.2024.28.pr6

**Affiliations:** Johns Hopkins University Bloomberg School of Public Health, United States

No accompanying comment.
